# Pharmacological vitamin C inhibits mTOR signaling and tumor growth by degrading Rictor and inducing HMOX1 expression

**DOI:** 10.1371/journal.pgen.1010629

**Published:** 2023-02-14

**Authors:** Senlin Qin, Guoyan Wang, Lei Chen, Huijun Geng, Yining Zheng, Chao Xia, Shengru Wu, Junhu Yao, Lu Deng

**Affiliations:** College of Animal Science and Technology, Northwest A&F University, Yangling, Shaanxi, China; Brigham and Women’s Hospital, UNITED STATES

## Abstract

Pharmacological vitamin C (VC) is a potential natural compound for cancer treatment. However, the mechanism underlying its antitumor effects remains unclear. In this study, we found that pharmacological VC significantly inhibits the mTOR (including mTORC1 and mTORC2) pathway activation and promotes GSK3-FBXW7-mediated Rictor ubiquitination and degradation by increasing the cellular ROS. Moreover, we identified that HMOX1 is a checkpoint for pharmacological-VC-mediated mTOR inactivation, and the deletion of FBXW7 or HMOX1 suppresses the regulation of pharmacological VC on mTOR activation, cell size, cell viability, and autophagy. More importantly, it was observed that the inhibition of mTOR by pharmacological VC supplementation *in vivo* produces positive therapeutic responses in tumor growth, while HMOX1 deficiency rescues the inhibitory effect of pharmacological VC on tumor growth. These results demonstrate that VC influences cellular activities and tumor growth by inhibiting the mTOR pathway through Rictor and HMOX1, which may have therapeutic potential for cancer treatment.

## Author summary

Vitamin C (VC) is an essential nutrient for human beings, and pharmacological VC is a potential natural compound for cancer treatment. The mTOR pathway plays an important role in tumor formation and progress, but whether VC can have an anticancer effect on the mTOR pathway or not remains unclear. In this study, we found that pharmacological VC blocked the activation of the mTOR pathway and tumor growth by degrading Rictor and inducing HMOX1 expression. Mechanistically, VC induced intracellular ROS, which can promote the expression of HMOX1 and GSK3-FBXW7-mediated ubiquitination of Rictor. Moreover, functional analysis demonstrated that pharmacological VC regulated the viability, autophagy, and apoptosis of cancer cells. Most importantly, the xenografted mouse models showed that VC acted as an anticancer agent by inhibiting the activity of the mTOR pathway. Thus, our study revealed a new anticancer mechanism of VC by inhibiting the mTOR pathway, thereby providing the evidence for the therapeutic potential of VC, when combined with other drugs, in cancer treatment.

## Introduction

Vitamin C (VC), being an essential micronutrient, plays an indispensable role in cellular oxidative metabolism through the scavenging of reactive oxygen species (ROS) [1]. VC is absorbed in the human intestine in two forms, as VC (ascorbate) or its oxidized form dehydroascorbate (DHA) [2], and is taken up through SVCT1/SVCT2 and GLUT1, respectively. The intracellular VC concentration is strictly regulated in order to maintain levels of 80–100 μM in the plasma [2, 3]. However, intravenous injection bypasses this strict regulation, allowing specific VC concentrations to be maintained within a specified period, thereby providing a pharmacological basis for its therapeutic application [4]. Several pioneering studies have demonstrated the efficacy of pharmacological VC in improving the survival of patients with advanced cancers [5, 6]. In contrast, two randomized double-blind controlled trials failed to demonstrate any benefit of VC against advanced malignant disease [7, 8]. Therefore, the route of administration is important for high-dose VC to have a therapeutic effect, and only intravenous administration results in sufficiently high plasma and urine concentrations to allow potential antitumor activity [9].

Recently, it has been demonstrated that VC at pharmacological plasma concentrations, acquired intravenously, can selectively kill KRAS- or BRAF-mutated colorectal cancer cells by targeting GAPDH [10]. Moreover, another study reported that VC preferentially kills hepatocellular cancer stem cells through SVCT2 [11]. Significantly, the results of several *in vivo* studies have provided further evidence for the antitumor efficacy of high-dose VC when administered parenterally [12, 13]. Mechanistically, pharmacological VC induces cancer cell death in a ROS-, AMPK-, and MAPK-dependent manner [14–17], while the toxicity of pharmacological VC can be completely inhibited *in vitro* by the enzymes that metabolize H_2_O_2_ [18–20]. Although VC was proposed to have a role to play in cell death over 40 years ago, the precise mechanism underlying this effect are yet to be elucidated.

The mechanistic target of rapamycin (mTOR) is a master regulator of metabolic homeostasis, and blocking mTOR activation through various rapalogs has proved to be an efficient therapeutic option in controlling a variety of diseases [21]. Notably, mTOR exists in two distinct multiprotein complexes, namely, mTOR complex 1 (mTORC1) and 2 (mTORC2). mTORC1 is composed of mTOR, which is the catalytic subunit, and four related proteins: the regulatory-associated protein of mTOR complex 1 (Raptor), proline-rich AKT substrate 40 kDa (PRAS40), DEP domain-containing mTOR-interacting protein (DEPTOR), and mammalian lethal with SEC13 protein 8 (mLST8) [22].

In addition to mTOR and mLST8, the core components of mTORC2 include the rapamycin-insensitive companion of mTOR (Rictor) and mammalian stress-activated protein kinase interacting protein 1 (mSIN1) [23]. mTORC1 regulates multiple physiological processes through phosphorylating S6 kinase 1 (S6K1) and eIF4E binding protein 1 (4EBP1), including cell viability, autophagy, ferroptosis, and apoptosis [21, 24]. mTORC2, in contrast, responds to the growth factor signaling through the phosphorylation of its downstream effectors AKT, PKC, and SGK1 [23]. Moreover, mTOR activation is precisely regulated by metabolic inputs such as signals related to amino acids and growth factors. Amino acids are essential activators of mTORC1 through RagA/B or C/D GTPase heterodimer complexes, which recruit mTORC1 to the lysosomal surface, while growth factors regulate the activation of lysosome-localized mTORC1 through the AKT-TSC-Rheb signal axis [21].

In the present study, we found that SVCT2-mediated uptake of VC increases intracellular ROS levels and regulates cellular activities by inhibiting the activation of the mTOR (including mTORC1 and mTORC2) pathway. Moreover, we found that pharmacological VC significantly inhibits the activation of the mTOR pathway. The results also demonstrated that pharmacological VC treatment leads to the ubiquitination of Rictor and, subsequently, mediates its degradation, while the treatment with the ROS scavenger *N*-acetylcysteine (NAC) has the potential to reverse pharmacological-VC-mediated Rictor degradation. Furthermore, the RNA-seq analysis determined that the exposure to pharmacological VC resulted in the upregulation of heme oxygenase 1 (HMOX1) expression, while the pharmacological VC administration failed to inhibit the mTOR activation in HMOX1-deficient cells, indicating that the regulation of HMOX1 expression represents another important mechanism underlying how pharmacological VC inhibits mTOR pathway activation. Additionally, we demonstrated that the elevated levels of VC in the plasma resulting from a high-dose intraperitoneal injection markedly inhibited the growth of xenografted tumors by suppressing mTOR activation, suggesting that the phenotype can be suppressed by HMOX1 knockdown. Thus, our study revealed a *de facto* regulatory mechanism by which pharmacological VC mediates the inhibition of mTOR activation, and, consequently, tumorigenesis.

## Results

### Pharmacological VC Inhibits mTOR Activity

**Fig 1:**
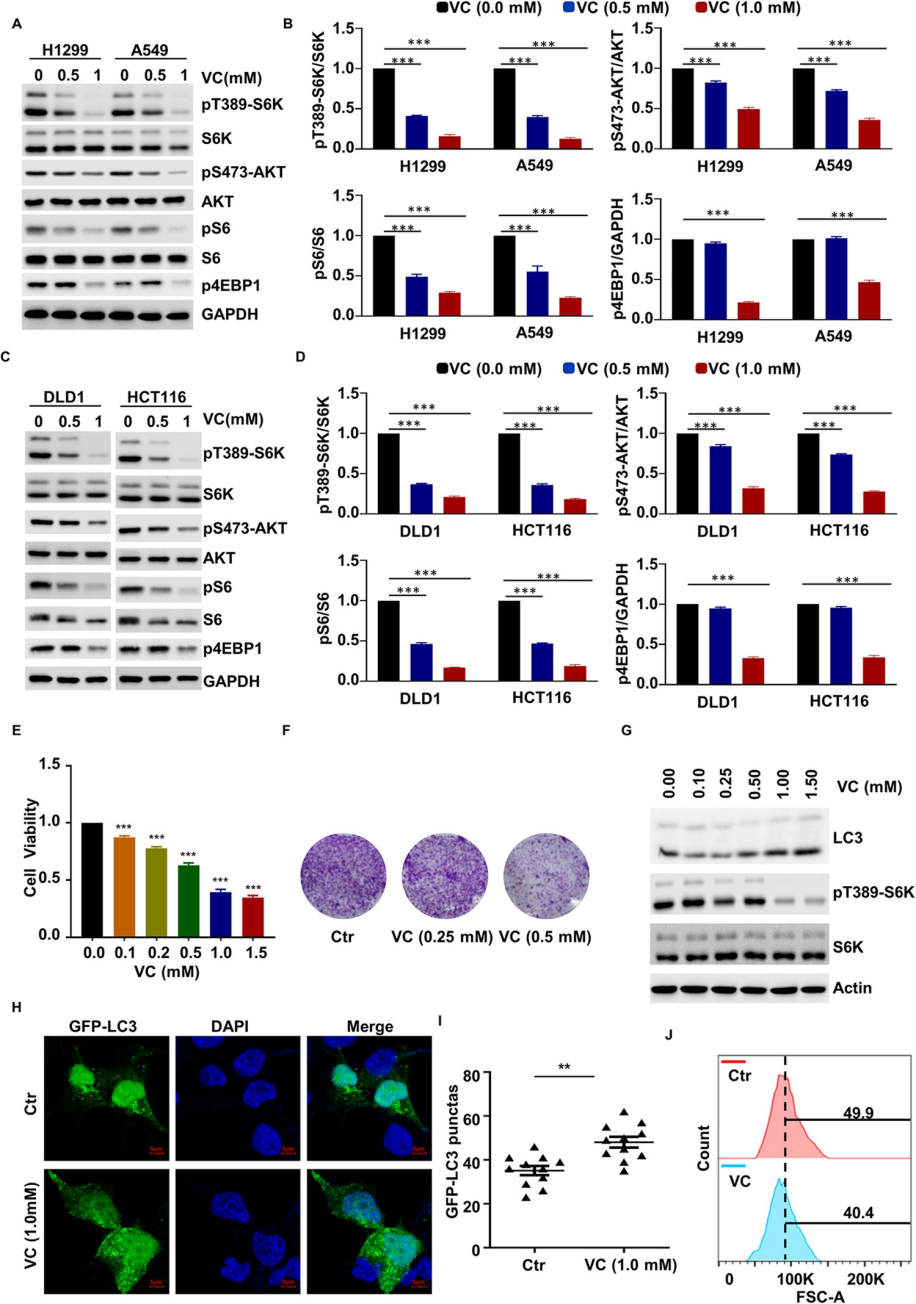
Pharmacological VC Inhibits mTOR Activity. (A-D) H1299/A549 (A, B) and DLD1/HCT116 (C, D) cells were treated with the indicated concentrations of VC for 2 h, the indicated proteins were detected by WB (A, C), and quantified by ImageJ, n = 3 (B, D). (E) H1299 cells were treated with the indicated concentrations of VC for 48 h, after which cell viability was detected by cell counting kit-8 (CCK-8), n = 3. (F) H1299 cells were treated with the indicated concentrations of VC following which cell viability was assessed by a clone formation assay. (G) H1299 cells were treated with the indicated concentrations of VC for 6 h after which autophagy was analyzed by examining LC3II levels. (H, I) H1299 cells were treated with bafilomycin A1 and 1 mM VC for 6 h, after which cell autophagy was analyzed by examining GFP-LC3 puncta (H), quantitative data for the GFP-LC3 puncta are presented (I). (J) H1299 cells were treated with 0.5 mM VC for 48 h and cell size was assessed by FACS. Data were analyzed by one-way ANOVA (B, D, E), or t-test (I), *p* value was considered statistically significant, ** denote *p* values of < 0.01, *** denote *p* values of < 0.001.

To explore the effect of pharmacological VC on mTOR pathway activation, we treated lung cancer cells (H1299 and A549) with different concentrations of VC for 2 hours and examined the activation status of mTORC1 by assessing the levels of S6K, S6, and 4EBP1 phosphorylation [25, 26]. The results showed that VC treatment reduced the pT389-S6K, pS6, and p4EBP1 levels in a dose-dependent manner, and, at the 1.0 mM concentration, completely abolished the mTORC1 activation (Fig 1A and 1B). Moreover, we also tested the activation of the mTORC2 pathway by evaluating the phosphorylation status of AKT at Ser473 and found that pharmacological VC significantly reduced the level of pS473-AKT in H1299 and A549 cells (Fig 1A and 1B). These results indicate that pharmacological VC blocks both the mTORC1 and mTORC2 pathways in a dose-dependent manner.

To examine whether the ability of pharmacological VC to inhibit the mTOR pathway is unique to lung cancer cells, we also treated colon cancer cells (DLD1 and HCT116) with different concentrations of pharmacological VC and again found that S6K, S6, 4EBP1, and AKT phosphorylation were inhibited (Fig 1C and 1D). Similar results were obtained for the cells of other tumor types, including HeLa (cervical cancer) and MDA-MB-231 (breast cancer) cells (S1A and S1B Fig), suggesting that pharmacological VC can inhibit the mTOR pathway in a variety of cancer cells. Furthermore, the intracellular concentration of VC was evaluated through high-performance liquid chromatography, which demonstrated that the intracellular VC content increased in a dose- and time-dependent manner (S1C and S1D Fig).

As mTOR is a key regulator of cell viability, we determined the effect of VC on the viability of the different cell lines using Cell Counting Kit-8 (CCK-8) assay and found that the viability of H1299 and HCT116 cells was significantly inhibited following 48 hours of pharmacological VC treatment. Moreover, the viability of H1299 and HCT116 cells was reduced by over 50% when the VC concentration reached 1.5 mM (Figs 1E and S1E), indicating that these effects were exerted in a concentration-dependent manner. Furthermore, we used a clone formation assay to assess the effect of pharmacological VC on cell viability and found that clone formation was inhibited in H1299 and HCT116 cells in a dose-dependent manner (Figs 1F and S1F). These data indicate that pharmacological VC inhibits the cell viability of cancer cells. Afterward, we measured the effect of pharmacological VC on autophagy, of which the mTOR pathway is a critical regulator. The data demonstrated that pharmacological VC treatment resulted in a sharp increase in the level of light chain 3 (LC3) II and a reduction in that of p62 in H1299 and HCT116 cells (Figs 1G and S1G). Additionally, the application of pharmacological VC led to the promotion of autophagy, as evidenced by an increase in the number of punctate GFP-LC3 foci following bafilomycin A1 treatment (Figs 1H, 1I, S1H and S1I). In order to determine whether the effects of pharmacological VC on mTOR signaling are physiologically significant, we also measured the effect of pharmacological VC on cell size and apoptosis, of which the mTOR pathway is a critical regulator. We found that the pharmacological-VC-treated cells were smaller than the control cells (Fig 1J) and that at the 1 mM concentration, pharmacological VC led to a significant increase in the rate of cell apoptosis in both H1299 and HCT116 cells (S1J–S1M Fig). Accordingly, we concluded that pharmacological VC is a negative regulator of mTOR-mediated cellular activities, including the control of cellular autophagy, cell size, and apoptosis.

### Pharmacological VC Affects Cell Size, Cell Viability, Autophagy, and Tumor Growth by Inhibiting mTOR Activation

**Fig 2:**
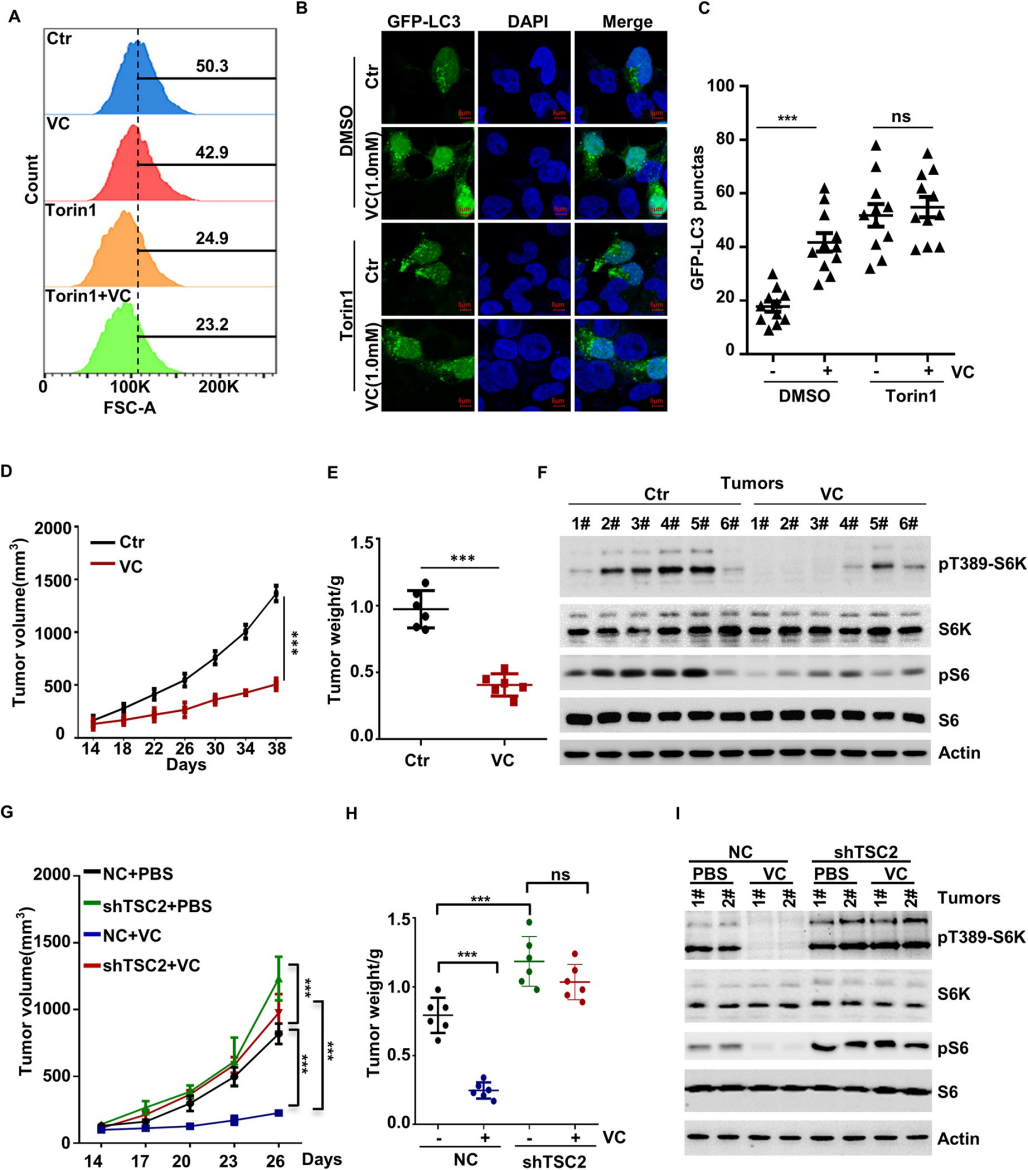
Pharmacological VC Affects Cell Size, Autophagy, and Tumor Growth through Inhibiting mTOR Activation. (A) H1299 cells were treated with 0.5 mM VC and Torin1 for 48 h, and cell size was determined by FACS. (B, C) H1299 cells were treated with bafilomycin A1 and 1.0 mM VC alone or in combination with Torin1 for 6 h, and then cell autophagy was analyzed by examining GFP-LC3 puncta (B). Quantitative data for GFP-LC3 puncta was presented (C). (D-F) H1299 were injected into nude mice subcutaneous, VC supplementation by intravenous injection (n = 6 per group). The diameter of the tumor was measured after 14 days of injection. Tumors were obtained on the 38th day after injection. The volume (D), weight (E), and mTORC1 activation (F) of tumors were measured. (G-I) TSC2-depleted H1299 cells were injected into nude mice subcutaneous, VC supplementation by intravenous injection (n = 6 per group). The diameter of the tumor was measured after 14 days of injection. Tumors were obtained on the 26th day after injection. The volume (G), weight (H), and mTORC1 activation (I) of tumors were measured. Data were analyzed by two-way ANOVA (C, D, G, H) or t-test (E), *p* value was considered statistically significant, *** denote p values of < 0.001, ns denote not significant.

To further confirm that the negative effect of pharmacological VC on the aforementioned cellular processes is indeed achieved by inhibiting mTOR activation, we treated the cells with the mTOR inhibitor Torin1, which blocks the activation of both mTORC1 and mTORC2. We found that pharmacological VC treatment did not further aggravate the inhibitory effects of Torin1 on cell size and cell viability (Figs 2A and S2A). In line with these results, we found that the effects of pharmacological VC on inhibiting cell viability can be blocked by MHY1485, the activator of mTORC1 (S2B Fig), which indicates that the negative effects of VC on these two processes are mediated through mTOR inhibition. Moreover, we found that pharmacological VC treatment did not further enhance Torin1-induced autophagy (Fig 2B and 2C).

Next, we sought to determine whether pharmacological VC is involved in mTOR-mediated tumorigenesis *in vivo*. To this end, H1299 cells were injected subcutaneously into the nude mouse, and VC (2g/kg/day) or PBS (control) was administered using intraperitoneal injection. As Fig 2D and 2E shows, the xenografted cells exhibited a strong ability to form rapidly growing tumors in the control group, whereas VC treatment significantly reduced the tumor growth. Moreover, compared to the control group, VC treatment resulted in a significant reduction in tumor volume and weight (Fig 2D and 2E). Furthermore, the WB results showed that pharmacological VC treatment significantly reduced the activation of mTORC1, as assessed by the levels of pT389-S6K (Fig 2F). Additionally, we constructed a stable cell line with TSC2 knockdown, which is the classical model that leads to constitutively active mTOR, and found that the tumor suppressive effect of VC could be eliminated by the knockdown of TSC2 (Fig 2G–2I). Altogether, these findings suggest that pharmacological VC regulates cell size, cell proliferation, autophagy, apoptosis, and tumor activity by inhibiting the activation of the mTOR pathway.

### SVCT2 Mediates the Uptake of VC to Inhibit the mTOR Pathway

Because mTOR activation is closely associated with environmental stimuli such as amino acid- and insulin-related signals, we explored whether pharmacological VC treatment could affect mTOR activation mediated by these two factors. The results demonstrated that both amino acid and insulin stimulation led to a significant increase in the levels of pT389-S6K and pS473-AKT (S3A and S3B Fig). Significantly, we found that pharmacological VC treatment could block the insulin-induced increase in the phosphorylation levels of S6K (S3A Fig) but did not affect the amino-acid-mediated activity (S3B Fig). Therefore, these findings suggest that pharmacological VC acts as a negative regulator of insulin-induced mTOR activation.

To further explore the mechanisms underlying the suppressive effect of pharmacological VC on mTORC1 activation, we examined whether pharmacological VC inhibits mTOR through AMPK or MAPK, reported to be the mechanism mediating the tumor-killing properties of pharmacological VC [16, 17]. We found that the siRNA knockdown of AMPK or p38 did not block the inhibitory effect of VC on the mTOR pathway and the treatment with neither an AMPK inhibitor (compound C) nor a MAPK inhibitor (SB203580) could restore the inhibitory effect of pharmacological VC on the pT389-S6K and pS473-AKT levels (S3C–S3F Fig), demonstrating that pharmacological VC inhibits the mTOR pathway independently of AMPK and MAPK.

**Fig 3:**
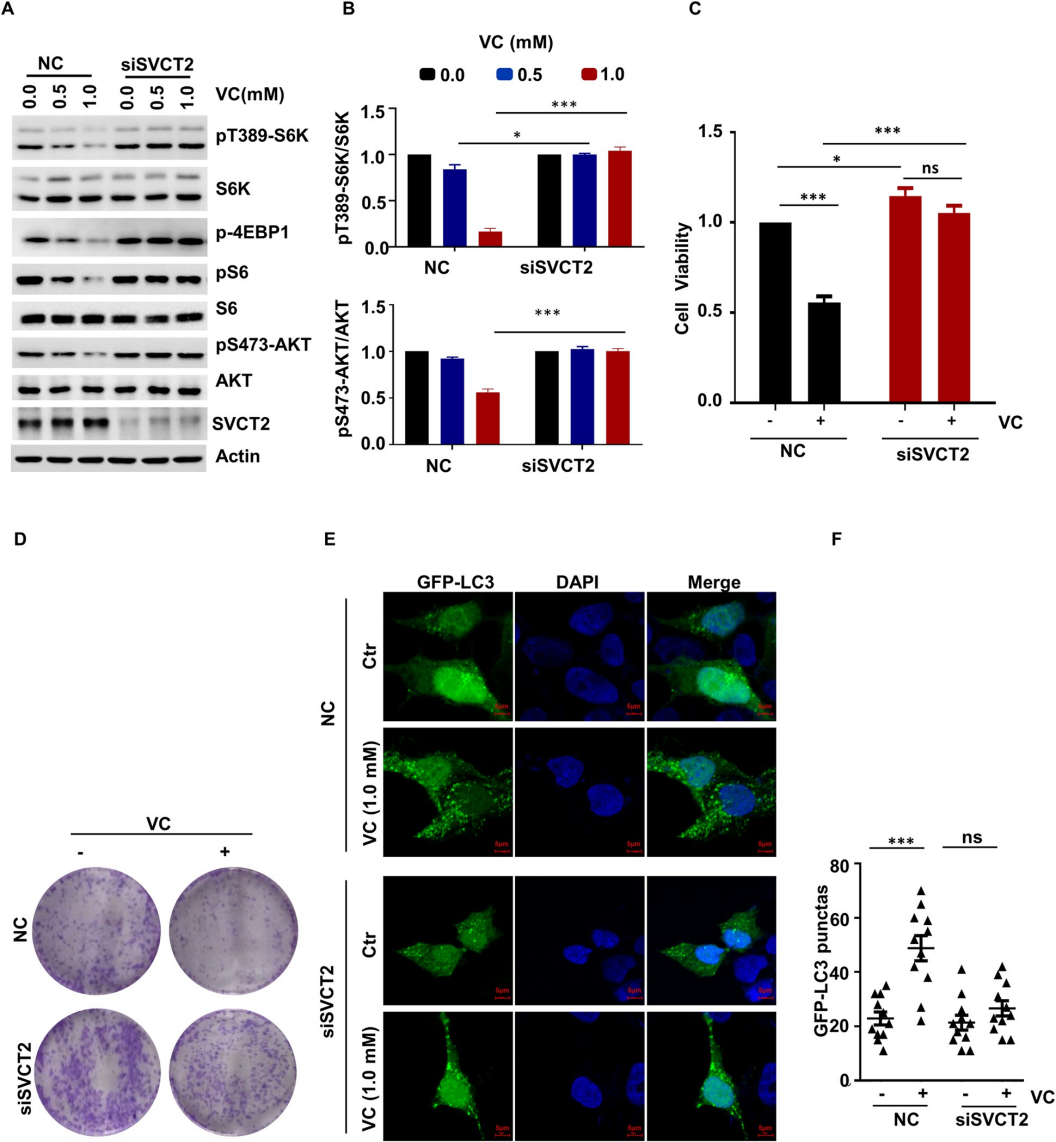
SVCT2 Mediates the Uptake of VC to Inhibit the mTOR Pathway. (A, B) SVCT2-knockdown H1299 cells were treated with the indicated concentrations of VC for 2 h, the indicated proteins were detected by WB (A), and quantified by ImageJ, n = 3 (B). (C, D) SVCT2-knockdown H1299 cells were treated with 1.0 mM VC, and cell viability was detected by CCK-8 (C) or clone formation assay (D). (E, F) SVCT2-depleted H1299 cells were treated with bafilomycin A1 and 1.0 mM VC for 6 h, and then cell autophagy was analyzed by examining GFP-LC3 puncta (E), quantitative data for GFP-LC3 puncta are presented (F). Data were analyzed by two-way ANOVA (B, C, F), *p* value was considered statistically significant, * denote *p* values of < 0.05, *** denote *p* values of < 0.001, ns denote not significant.

The VC transport is mediated by SVCT family members (i.e., SVCT1 and 2) in a Na^+^-dependent manner, while the uptake of DHA (i.e., the oxidized form of VC) is accomplished by GLUT1 and Na^+^-independent facilitated diffusion [2]. The results of our study showed that STF-31, a GLUT1 inhibitor, did not affect the ability of pharmacological VC to inhibit mTOR activation (S3G and S3H Fig). Consequently, we hypothesized that pharmacological-VC-mediated mTOR inactivation may be achieved through SVCT1 or SVCT2. To confirm this, we knocked down SVCT1 or SVCT2 using specific siRNAs and found that the knockdown of SVCT2, but not SVCT1, rescued the pharmacological-VC-mediated inhibition of mTOR activation (Figs 3A, 3B, S3I and S3J). Collectively, these results indicate that SVCT2-mediated VC uptake regulates mTOR activation in a GLUT1-independent manner.

Next, we detected the effects of SVCT2 in cell proliferation. In comparison with the WT cells, SVCT2 silencing abrogates the inhibitory effect of VC on cell viability (Fig 3C and 3D). Notably, the depletion of SVCT2 also significantly suppressed the pharmacological-VC-mediated autophagy (Fig 3E and 3F).

### Pharmacological VC Inhibits the mTOR Pathway in a ROS-dependent Manner

**Fig 4:**
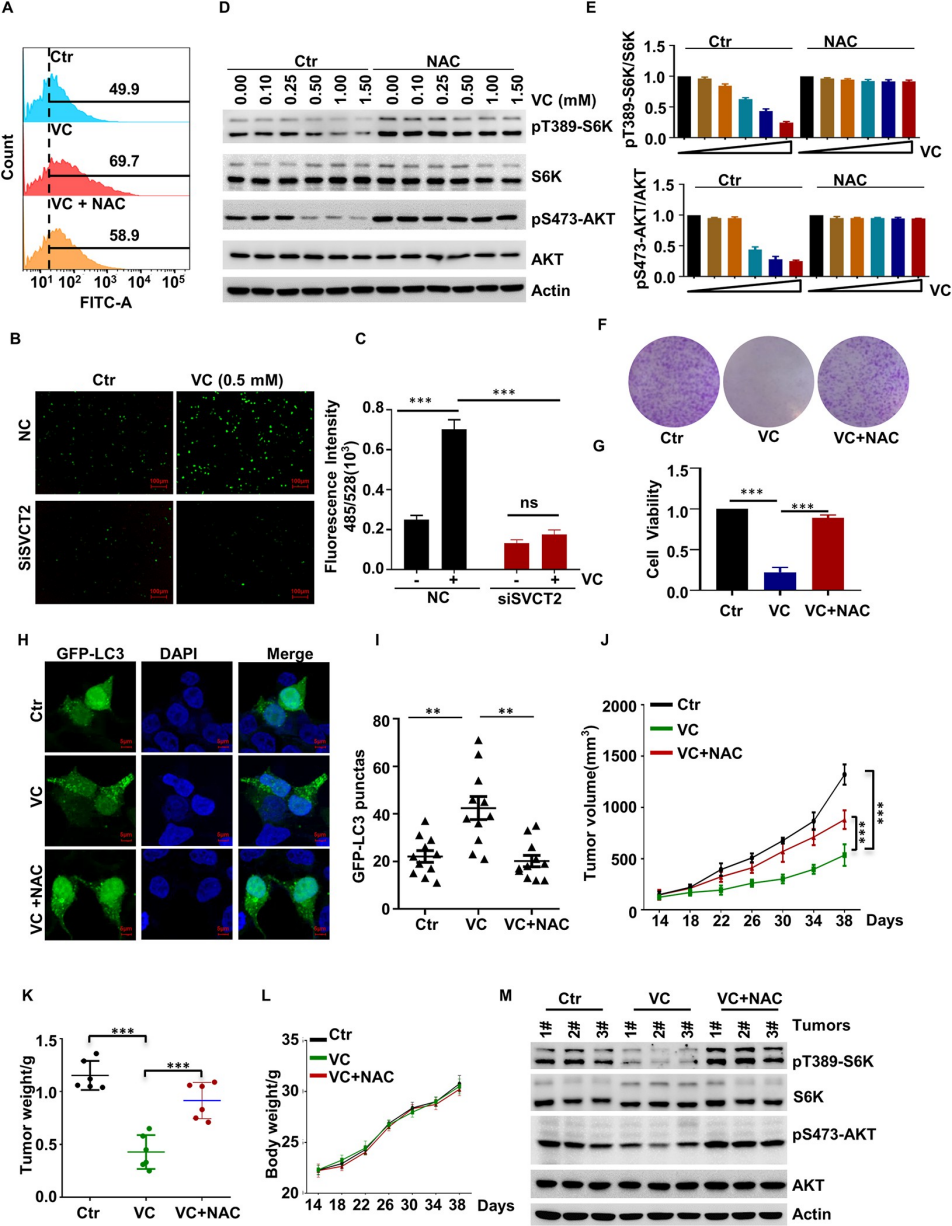
Pharmacological VC to Inhibit the mTOR Pathway in a ROS-dependent Manner. (A) H1299 cells were treated with 1.0 mM VC alone or in combination with 2.0 mM *N*-acetylcysteine (NAC), and the ROS levels in the indicated cells were detected by FACS. (B, C) SVCT2-knockdown H1299 cells was treated with 0.5 mM VC, and the ROS of cells was detected by microplate-reader (B), the quantified data of fluorescence intensity were present, n = 3 (C). (D, E) H1299 cells were treated with the indicated concentrations of VC alone or in combination with 2.0 mM NAC for 2 h, the indicated proteins were detected by WB (D), and quantified by ImageJ, n = 3 (E). (F, G) H1299 cells were treated with 0.5mM VC alone or in combination with 2.0 mM NAC, and following which cell viability was assessed by clone formation (F) or CCK-8 (G) assay, n = 3. (H, I) H1299 cells were treated with bafilomycin A1 and 1.0 mM VC alone or in combination with NAC for 6 h, after which cell autophagy was analyzed by examining GFP-LC3 puncta (H), quantitative data for the GFP-LC3 puncta are presented (I). (J-M) H1299 cells were injected into nude mice subcutaneous, VC supplementation by intravenous injection, NAC was administered to the mouse (1g/L drinking water, pH 7) and throughout the duration of experiment (n = 6 per group). The diameter of the tumor was measured after 14 days of injection. Tumors were obtained on the 38th day after injection. The tumors volume (J), tumors weight (K), mice weight (L), and mTORC1 activation in tumor samples (M) was measured. Data were analyzed by one-way ANOVA (G, I, K) or two-way ANOVA (C, J, L), *p* value was considered statistically significant, ** denote *p* values of < 0.01, *** denote *p* values of < 0.001, ns denote not significant.

Given that VC treatment mainly kills cells by enhancing the endogenous ROS production, we treated the cells with VC for 2 hours before measuring the cellular ROS levels by using dichlorofluorescein (DCF) staining. As expected, the pharmacological VC treatment significantly increased the cellular ROS levels (Figs 4A, S4A and S4B). Afterward, we examined whether SVCT2 has any effect on pharmacological VC-induced ROS, observing an increase in the cellular ROS levels after pharmacological VC treatment in the WT cells but not in the SVCT2-deficient cells (Fig 4B and 4C). In order to determine whether the effect of pharmacological VC on the mTOR pathway is exerted through increased ROS generation, we treated the cells with 2 mM of the ROS scavenger NAC and found that NAC could restore the mTOR pathway activation in H1299 cells treated with pharmacological VC (Figs 4D, 4E and S4C). Next, we examined whether pharmacological-VC-induced ROS production affects mTOR-mediated cell viability. Indeed, the results showed that the inhibitory effect of pharmacological VC on the viability of H1299 and HCT116 cells can be abolished through NAC treatment (Figs 4F, 4G and S4D), which suggest that the pharmacological VC-mediated suppression of mTOR activation reduces cell viability in a ROS-dependent manner. Moreover, we also found that the promoting effect of pharmacological VC on autophagy in H1299 cells could be decreased through pretreatment with NAC (Fig 4H and 4I). More importantly, we found that NAC blocked the inhibitory effect of VC on tumor growth and mTOR activation by administrating NAC (1 g/L drinking water, pH 7) to mice throughout the duration of the experiment and no effect on body weight of mice (Fig 4J–4M).

Collectively, these results indicate that SVCT2 mediates the uptake of VC, which, subsequently, leads to the inhibition of the mTOR pathway in a ROS-dependent manner, thereby promoting autophagy and the suppression of cell size and tumor growth.

### Pharmacological VC Inhibits the mTORC1 Pathway by Promoting the Degradation of Rictor

**Fig 5:**
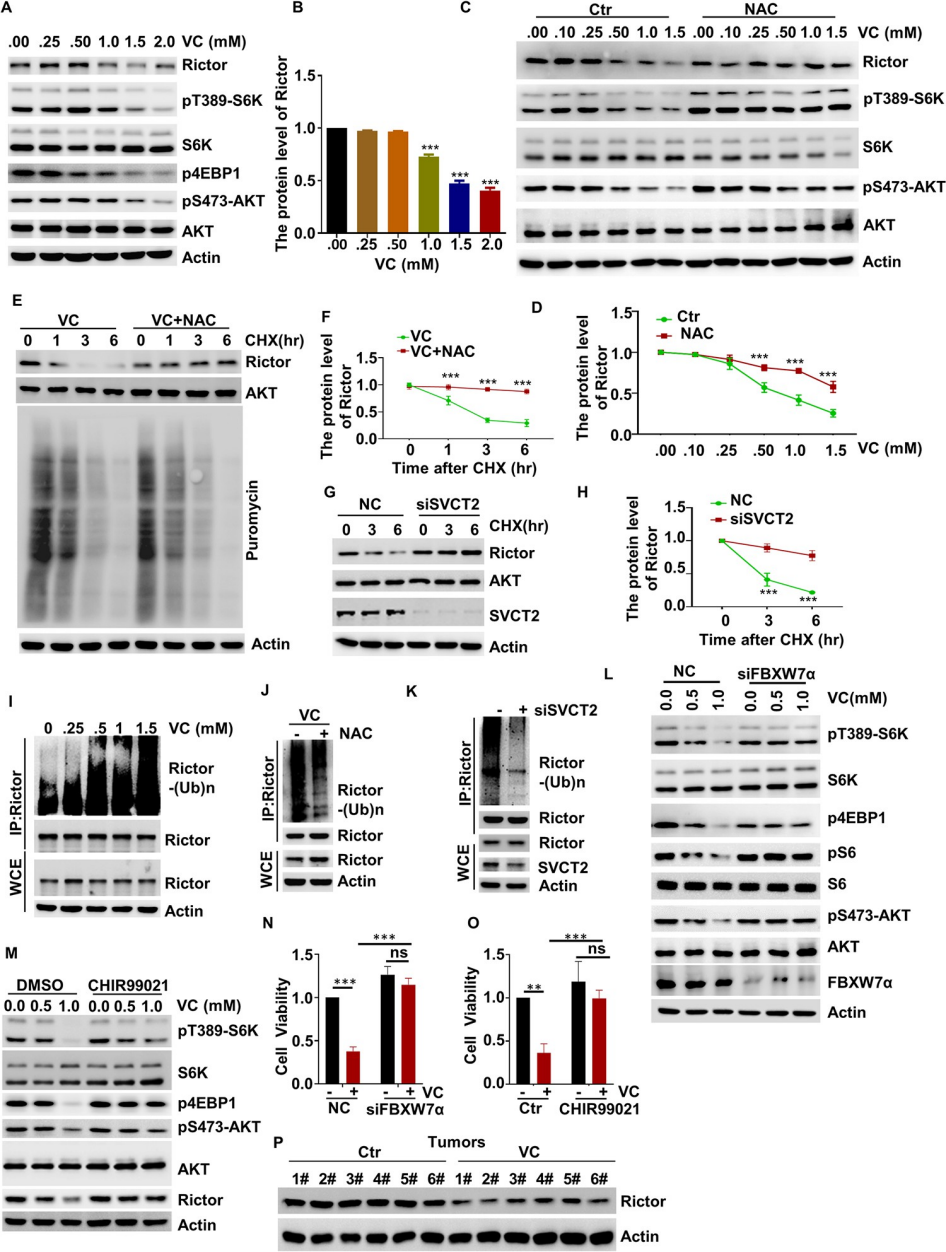
Pharmacological VC inhibits the mTORC1 pathway by promoting the degradation of Rictor. (A, B) H1299 cells were treated with the indicated concentrations of VC for 6 h, and then the levels of the indicated proteins were evaluated by WB (A). Quantitative data for Rictor protein level was presented, n = 3 (B). (C, D) H1299 cells were treated with the indicated concentrations of VC or NAC for 6 h, after which WB was used to evaluate the levels of the indicated proteins (C). Quantitative data for Rictor protein level was presented, n = 3 (D). (E, F) H1299 cells were incubated with puromycin (1 μM) and VC or NAC for 6 h. At the indicated time points, CHX was added and after which WB was used to evaluate the levels of the indicated proteins (E). Quantitative data for Rictor protein level was presented, n = 3 (F). (G, H) SVCT2-knockdown H1299 cells were treated with CHX for the indicated time, and then WB was used to evaluate the levels of the indicated proteins (G). Quantitative data for Rictor protein level was presented, n = 3 (H). (I) H1299 cells were treated with the indicated concentrations of VC for 6 h, and the ubiquitination of Rictor was evaluated by WB. (J) H1299 cells were treated with VC or NAC for 6 h, and the ubiquitination of Rictor was evaluated by WB. (K) The ubiquitination of Rictor was evaluated in SVCT2-knockdown H1299 cells. (L) FBXW7α-knockdown H1299 cells were treated with the indicated concentrations of VC for 2 h, the indicated proteins were detected by WB. (M) H1299 cells were treated with the indicated concentrations of VC alone or in combination with CHIR99021 for 6 h, the indicated proteins were detected by WB. (N) FBXW7α-knockdown H1299 cells treated with 0.5 mM VC, and then cell viability was assessed by CCK-8 assay, n = 3. (O) H1299 cells were treated with 0.5 mM VC alone or in combination with CHIR99021, and then cell viability was assessed by CCK-8 assay, n = 3. (P) The Rictor was tested in the tumor samples via WB. Data were analyzed by one-way ANOVA (B) or two-way ANOVA (D, F, H, N, O), *p* value was considered statistically significant, ** denote *p* values of < 0.01, *** denote *p* values of < 0.001, ns denote not significant.

In this study, we sought to further elucidate the molecular mechanisms through which mTOR activity is inhibited by pharmacological VC in a ROS-dependent manner. As mTORC2 acts upstream of mTORC1 [22], we first measured the protein component levels of the mTORC2 complex. We found that VC treatment led to a significant decrease in Rictor protein levels (Fig 5A and 5B), and the degradation of Rictor was significantly suppressed through pretreatment with NAC (Fig 5C and 5D). In contrast, the levels of Rictor transcripts remained relatively stable in response to pharmacological VC treatment (S5A Fig), indicating that pharmacological VC regulates the protein level of Rictor at the posttranscriptional level.

Moreover, in order to further illustrate that pharmacological VC promotes the degradation of Rictor, we co-treated H1299 cells with VC and cycloheximide (CHX), which blocks new protein synthesis, and determined its effect on Rictor stability. As S5B and S5C Fig shows, compared to control cells, pretreatment with pharmacological VC significantly increased CHX-mediated Rictor instability; however, this effect was abolished by NAC treatment (Fig 5E and 5F), indicating that pharmacological VC promotes the instability of Rictor in a ROS-dependent manner. Additionally, the half-life of Rictor was dramatically delayed in SVCT2-deficient cells (Fig 5G and 5H). Furthermore, to examine whether the degradation of Rictor is dependent on the ubiquitin/proteasome system, we treated H1299 cells with the 26S proteasome inhibitor MG132 at the indicated time point. We found that Rictor degradation was blocked following MG132 treatment (S5D and S5E Fig), indicating that the degradation of Rictor downstream of VC treatment was mainly due to ubiquitination. Consistently, pharmacological VC treatment significantly enhanced the ubiquitination of Rictor (Fig 5I), while the ubiquitination of Rictor significantly decreased after NAC treatment or in SVCT2-deficient cells (Fig 5J and 5K), which suggests that pharmacological VC-dependent Rictor degradation is mainly caused by ROS and ubiquitination. Altogether, these findings suggest that pharmacological VC promotes Rictor proteolysis through the ubiquitin–proteasome degradation pathway.

### Pharmacological VC Promotes the Degradation of Rictor through GSK3-FBXW7

The stability of Rictor has been reported to be regulated by FBXW7α [27], which is consistent with our finding that the pharmacological-VC-mediated mTOR inactivation was rescued in the FBXW7α knockdown cells (Fig 5L). Moreover, previous studies found that Rictor is degraded through an FBXW7α-mediated ubiquitination in a GSK3-dependent manner [27]. Thus, we examined whether GSK3 had any effect on Rictor degradation and mTORC1 activation and found that LiCl and CHIR99021, the inhibitors of GSK3, markedly increased the protein levels of Rictor in response to pharmacological VC treatment (Figs 5M, S5F and S5G). Meanwhile, we also examined whether GSK3 affected mTOR activation and found that pharmacological VC was unable to suppress the phosphorylation of S6K1, 4EBP1, and AKT in LiCl- and CHIR99021-treated cells (Figs 5M, S5H and S5I). In addition, the inhibitory effects of pharmacological VC on cell proliferation were counteracted by treatment with FBXW7α siRNA, CHIR99021, and LiCl (Figs 5N, 5O and S5J). Furthermore, while verifying the related mechanism, we found a decrease in the protein level of Rictor in the pharmacological-VC-supplemented tumor samples (Fig 5P), indicating the necessity of GSK3-FBXW7-induced Rictor degradation in the pharmacological-VC-dependent mTOR inactivation.

### Pharmacological VC inhibits the mTORC1 pathway by inducing HMOX1 expression

**Fig 6:**
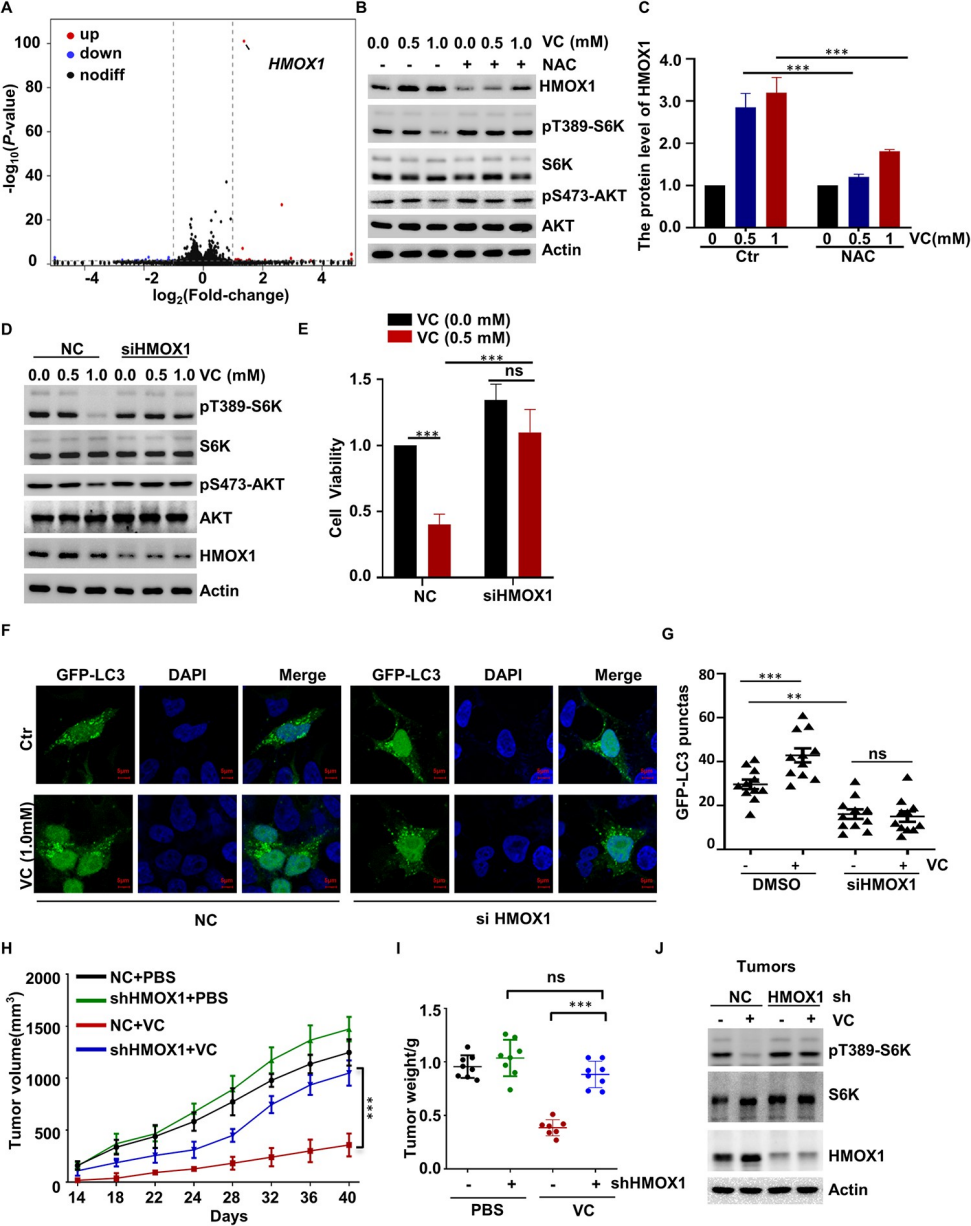
Pharmacological VC inhibits the mTORC1 pathway by inducing HMOX1 expression. (A) Volcano plot of differentially expressed genes (DEGs) between VC-treated and control (Ctr) cells. (B) H1299 cells were treated with VC alone or in combination with NAC for 2 h, the expression of HMOX1 was detected by WB. (C) H1299 cells were treated with the indicated concentrations of VC alone or in combination with 2.0 mM NAC, and following which cell viability was assessed by CCK-8 assay, n = 3. (D) HMOX1-depleted H1299 cells were subjected to WB analysis to evaluate the levels of indicated proteins. (E) HMOX1-knockdown H1299 cells treated with 0.5 mM VC, and then cell viability was assessed by CCK-8 assay, n = 3. (F, G) The HMOX1-depleted H1299 cells were treated with bafilomycin A1 and 1.0 mM VC for 6 h, after which cell autophagy was analyzed by examining GFP-LC3 puncta (F) Quantitative data for the GFP-LC3 puncta are present (G) (H-J) HMOX1-depleted H1299 were injected into nude mice subcutaneous, VC supplementation by intravenous injection (n = 8 per group). The diameter of the tumor was measured after 14 days of injection. The tumor volume (H), weight (I), and mTORC1 activation (J) in tumor samples were measured. Data were analyzed by two-way ANOVA (C, E, G, H, I), *p* value was considered statistically significant, *** denote *p* values of < 0.001, ns denote not significant.

Next, we elucidated the molecular mechanisms underlying pharmacological-VC-induced mTOR inactivation through RNA-sequencing. Interestingly, we found that HMOX1 was significantly induced in response to pharmacological VC stimulation (Fig 6A). These results were further confirmed by qPCR and western blotting analyses (Figs 6B, 6C and S6A). Moreover, we found that the pharmacological-VC-mediated induction of HMOX1 expression was blocked by NAC treatment (Fig 6B and 6C), indicating that this effect was exerted in a ROS-dependent manner. Afterward, we examined the role of HMOX1 in regulating pharmacological-VC-dependent mTORC1 inhibition and found that pharmacological VC could not suppress the phosphorylation of S6K1 or AKT in the HMOX1-deficient cells (Fig 6D). Notably, we also observed that the treatment with the HMOX1 inhibitor ZnPPIX significantly reversed the suppressive effect of pharmacological VC on mTOR activation, indicating that HMOX1 is required for this process (S6B and S6C Fig).

Subsequently, we examined whether HMOX1 is involved in the pharmacological-VC-mediated regulation of cell proliferation and autophagy and found that the negative effects of pharmacological VC on cell viability were mostly abolished in the HMOX1-deficient cells (Fig 6E). Additionally, the HMOX1 inhibitor ZnPPIX significantly blocked the inhibitory effect of pharmacological VC on cell viability (S6D Fig). Later, we detected the effects of HMOX1 in pharmacological-VC-induced autophagy and found that ZnPPIX suppressed the autophagy induced by pharmacological VC treatment (S6E and S6F Fig). Moreover, compared to the WT cells, the deletion of HMOX1 significantly abolished the promoting effects on autophagy induced by pharmacological VC (Fig 6F and 6G).

Considering that the inhibition of mTOR could inhibit tumor growth, we explored whether the induction of mTORC1 inactivation could function as a potential therapeutic method for cancer treatment. To this end, we examined whether HMOX1 is involved in mTOR-mediated tumorigenesis *in vivo*. For this, HMOX1-deficient H1299 cells were injected into nude mice subcutaneously, and we treated the nude mice with or without pharmacological VC supplementation. The obtained data confirmed that the pharmacological VC supplementation blocked tumor growth (Fig 6H–6J). In addition, our data showed that pharmacological VC fails to inhibit the ability to form tumor with a rapid growth rate, as well as the phosphorylation of S6K in HMOX1-silenced cells (Fig 6H–6J). Thus, we suppose that the anti-cancer effects of pharmacological VC supplementation are at least partially attributable to HMOX1, which leads to the pharmacological-VC -dependent mTORC1 inactivation.

Altogether, these results demonstrate for the first time that pharmacological VC can inhibit the mTOR pathway by inducing the degradation of Rictor and upregulating the expression of HMOX1, thereby negatively influencing cell proliferation, cell size, autophagy, and tumorigenesis.

## Discussion

Ever since it was first reported that intravenous VC administration (10 g day^−1^) could effectively prolong the survival of cancer patients, the antitumor efficacy of VC has been the subject of intense debate [5, 7, 17]. According to a recent study, VC can selectively kill KRAS/BRAF-mutated colorectal cancer cells by targeting GAPDH [10]. In the present study, we found that pharmacological VC treatment can inhibit cell viability regardless of the BRAF mutation status by inhibiting the activation of the mTOR pathway [10]. Moreover, the obtained data suggest that pharmacological VC-mediated degradation of Rictor and regulation of HMOX1 expression represents an innovative mechanism underlying how VC regulates mTOR pathway activation and, consequently, autophagy, cell viability, and cell size.

Given the central role played by the mTORC1 and mTORC2 pathways under both physiological and pathological conditions [21], we investigated the effect of pharmacological VC treatment on these two pathways. The results showed that pharmacological VC can reduce the phosphorylation levels of S6K, S6, 4EBP1, and AKT in the tumor cells in a ROS-dependent manner. Several studies have reported that pharmacological VC treatment reduces ERK phosphorylation and inhibits the MAPK/ERK pathway in a ROS-dependent manner, however, the changes of p-p38 and p-AMPK were not detected in our experiments, probably because the cell lines we used (HCT116, H1299, A549, DLD1, HeLa, MDA-MB-231) were inconsistent with previous research (including ovarian cancer cells and thyroid cancer cells) [16, 17, 28]. Moreover, pharmacological VC has also been suggested to regulate cell viability and apoptosis through AMPK or MAPK [16, 17]; however, in our research, neither the AMPK inhibitor nor the MAPK inhibitor could attenuate the inhibitory effect of pharmacological VC on the mTORC1 and mTORC2 pathways, indicating that VC inhibits the mTOR pathway independently of AMPK and MAPK. Moreover, several studies have reported that pharmacological VC treatment activates rather than inhibits the mTORC1 and mTORC2 pathways [29, 30]. We suspect that the differences in the VC concentrations and cell lines used may contribute to this discrepancy. Significantly, we found that the inhibitory effect of pharmacological VC on the mTOR pathway was also dependent on upstream signals. In addition, pharmacological VC treatment significantly inhibited mTOR pathway activation, which may also help explain the differences between the findings reported in previous studies and our study.

In this study, we observed that pharmacological VC treatment could significantly reduce the Rictor levels. After conducting a more detailed analysis, we found that pharmacological VC treatment, instead of affecting the Rictor transcript levels, promoted the degradation of Rictor through the 26S proteasome. However, the specific mechanism underlying the VC-induced degradation of Rictor requires further investigation. For instance, the stability of Rictor has been reported to be regulated by FBXW7α [27], but it remains unknown whether pharmacological VC can regulate FBXW7α expression or the binding of Rictor to FBXW7α. Moreover, while ROS can reportedly also upregulate MUL1 expression and induce AKT degradation in different types of cancer [16, 31, 32], in our study, we did not find that pharmacological VC treatment could promote AKT degradation. The differences in the VC treatment duration may explain these contradictory observations, as in our study, VC treatment lasted for 2 hours, while in Su’s study, CHX treatment was administered for 60 minutes. The use of different cell lines may also be an important factor in this discrepancy.

Previous studies have suggested that various intra- and extracellular stimuli are capable of activating MAPK, PKC, AMPK and PI3K/Akt signaling pathways and inducing the expression of HMOX1 through Nrf2. Meanwhile, transcription factors, including activator protein-1 (AP-1), yin-yang 1 (YY1), STAT3, and HIF-1α induce HMOX1 expression through oxidative stress, hypoxia, heme, and IL-6 [33]. VC also promotes DNA demethylation and gene expression through the activation of TET proteins [34]. Similarly, Choudhury et al. found that although increased TET activity may lead to genome-wide hypomethylation and HMOX1 expression, they did not observe any significant changes in the promoter methylation of the HMOX1 gene [35]. Oxidative stress is an important factor in the induction of HMOX1 expression by a mechanism that is at least partially through Nrf2 [33]. Notably, many studies have added catalase, which converts H_2_O_2_ to water, and/or L-ascorbic acid 2-phosphate, which is a derivative of VC that is not easily oxidized, to their experiments in order to exclude oxidative stress as a potential mechanism [36–38]. In our study, we found that pharmaceutical VC significantly increased the expression of HMOX1 and cellular ROS levels. Moreover, we also found that while the NAC was able to inhibit the VC-mediated upregulation of HMOX1, it did not totally inhibit the upregulation of HMOX1, which may be caused by the fact that NAC did not completely scavenge ROS. Therefore, we suggest that the upregulation of HMOX1 may be mediated through Nrf2 in a ROS-dependent manner.

DHA, which is the oxidized form of VC, can be absorbed through GLUT1 and reduced to VC through the activity of glutathione (GSH), leading to ROS generation and accumulation. Previous studies have demonstrated that increased ROS levels result in the loss of GAPDH activity and subsequent ATP depletion, which ultimately inhibits cell viability [10]. The results obtained in our study show that the inhibitor of GLUT1 does not block the inhibitory effect of VC on the mTOR pathway. Moreover, we found that knockdown of SVCT2, but not SVCT1, neutralized the inhibitory effect of VC on mTOR activation. Therefore, we can conclude that VC enters the cell through SVCT2, which in turn regulates the mTOR pathway, while the oxidized form of VC, i.e., DHA, does not play an important role in the mTOR pathway, or, at the very least, DHA does not act on mTOR through GLUT1. The findings of a study conducted by Lv et al. suggest that VC enters the cell through SVCT2, thereby increasing intracellular ROS levels and causing DNA damage, energy exhaustion, and, ultimately, the apoptosis of liver cancer stem cells [11]. Consistent with this, the results of our study showed that the lack of SVCT2 reversed the inhibitory effect of VC on the mTOR pathway. Notably, we found that SVCT2-mediated uptake of VC can significantly inhibit mTOR pathway activation, which may also be one of the mechanisms by which VC preferentially eliminates liver cancer stem cells.

Furthermore, our study investigated that VC treatment induced autophagy and, thus, inhibited cancer cells viability, which may play a role in early tumorigenesis. The dysregulation of autophagy has been found in various diseases, including neurodegenerative diseases, inflammatory diseases, and tumors [39], but autophagy is a double-edged sword, and in late-stage tumors, it can act as a dynamic degradation and recycling system that contributes to tumor survival, growth, and metastasis [40]. Even so, in the early stages of tumorigenesis, autophagy as a cell death pathway can prevent tumor initiation and suppresses cancer progression. For example, mice with the deletion of the autophagy gene Beclin 1 develop spontaneous tumors, and patients with breast, ovarian, and prostate cancers also show harbor monoallelic loss of Beclin 1 [41, 42]. Meanwhile, 6-bromo-5-trans-4-methoxybenzaldehyde, T-oligas, and 6-azauridine (6-AZ) significantly upregulate autophagic activity, which induces autophagic death of tumor cells [43–45]. Therefore, autophagy induction by VC in various malignant cell lines may exert its anticancer activity, and the application of autophagy inducers to stimulate excessive tumor cell death may become a new avenue for future cancer prevention and treatment.

VC has been shown to have the ability to kill a variety of tumor cell types. The results of our study demonstrated that exposure to pharmacological VC reduced the viability of cancer cells. Pharmacokinetic studies have shown that intravenous injection of 4 g kg^−1^ of VC can yield a plasma concentration close to 2 mM, and our *in vitro* studies showed that this is sufficient to kill lung cancer cells. Considering the welfare of the mice and clinical availability, we adjusted the concentration to 2g/kg/day. Although this concentration is still slightly higher than the concentration achieved by patient treatment, through the mouse *in vitro* tumor-bearing model, we found that VC treatment was able to significantly inhibit tumor growth, even if it did not affect the body weight of the mice. These results indicate that VC can be used as a potential adjuvant therapy for the treatment of lung cancer. Several clinical trials have demonstrated that high-dose intravenous VC can improve the quality of life of patients with advanced cancer and prolong overall survival [46].

In this study, the mice are Gulo wild-type, which synthesize their own VC. However, under normal conditions, the concentration of VC in the serum of mice did not reach the pharmacological concentration of VC injecting intraperitoneally. As our title indicates, the concentration of VC we used was pharmacological, not physiological. Moreover, our findings suggest that the treatment with pharmacological concentrations of VC can significantly inhibit the growth of tumors. Given that *in vitro* conditions cannot fully recapitulate the conditions associated with hypoxia, hypoglycemia, or other metabolic changes in the body, more clinical trials are needed to further evaluate the potential clinical application of VC in tumor treatment and determine its safety and effectiveness.

Many previous studies have focused on the use of VC in combination with other targeted agents. For example, the addition of VC may enhance the efficacy of combined anti-CTLA-4 and anti-PD-1 blockade against tumors [47, 48], while i*n vitro* studies have found that high doses of VC may target mitochondria, which in turn leads to cancer stem cell death [49]. Enhanced therapeutic efficacy has also been confirmed by the combination of VC with telomerase inhibitors (triethylenetetramine), respiratory chain complex I inhibitors (metformin), ATP synthase inhibitors (oligomycin A) and Bcl-2 inhibitors (venetoclax) [50, 51]. Moreover, preclinical studies have found that high-dose VC has synergistic anticancer effects when combined with kinase inhibitors (sorafenib) [52] and EGFR inhibitors (cetuximab and gefitinib) [53, 54]. All of these studies illustrate the promise of VC in the co-induction of synergistic cancer cell killing. Furthermore, it is well recognized that the relentless activity of mTOR represents a major mechanism of resistance to targeted therapy, and there are many reports on the combined trials of mTOR inhibitor rapamycin with the aforementioned targeted agents [55–58]. The data obtained in this study suggest a potential mechanism by which VC induces cancer cell death by inhibiting the mTOR signaling pathway. To sum up, we believe that assessing whether VC can replace rapamycin (an inhibitor of the mTOR signaling pathway) to a certain degree for the treatment of specific tumors in relevant mouse models or replace rapamycin in combination with multiple drugs in order to induce the synergistic killing of cancer cells would be worthwhile for future studies.

In summary, the results of our study revealed an effective regulatory system underlying the pharmacological-VC-mediated suppression of cell viability, which is in turn mediated through the regulated activation of mTORC1 and mTORC2. These effects support the antitumor activity of pharmacological VC (Graphical Abstract). Thus, our study provides an unexpected model for controlling mTOR overactivation through pharmacological VC treatment and offers a potential therapeutic method for cancer treatment.

## Material and methods

### Ethics statement

All the animal-based experiments were performed in compliance with the Guide for the Care and Use of Laboratory Animals and were approved by the Institution Animal Care and Use Committee of the Northwest A&F University (NWAFU-2020-1131).

### Reagents

N-acetylcysteine (NAC, A7250), cycloheximide (CHX, 5.08739), dichlorofluorescein diacetate (DCF-DA) (35845), Torin1 (475991), ferrostatin-1 (Ferr-1, SML0583), deferoxamine (DFO, D9533), and MG132 (M7449) were obtained from Sigma-Aldrich (Missouri, USA); SB203580 (S1076), bafilomycin A1 (S1413), and compound C (S7306) were obtained from Selleck Chemicals (USA); STF-31 (4484) was obtained from TOCRIS (USA); ZnPPIX (14483) was purchased from Cayman Chemical; DMEM, RPMI 1640 medium, FBS, amino acids (50X), β-mercaptoethanol, penicillin, and streptomycin were purchased from Gibco (Utah, USA); DMEM (amino acid-free) was purchased from Genetimes Technology (Shanghai, China); the Cell Counting Kit-8 (CCK-8) (K009) was purchased from ZETA LIFE (CA, USA); vitamin C (VC) (ST1434) and puromycin dihydrochloride (ST551) were purchased from Beyotime Biotechnology (Shanghai, China); YF 488-Annexin V/PI Apoptosis Detection Kit (Y6002M) was purchased from US Everbright (Suzhou, China); and TRIzol reagent, PrimeScript RT reagent Kit (RR047A), and TB Green quantitative real-time quantitative polymerase chain reaction (qRT-PCR) kit (RR820A) were purchased from Takara (Dalian, China).

### Cell culture

H1299, HCT116, DLD1, A549, HeLa, and MDA-MB-231 were purchased from National Science & Technology Infrastructure (NSTI, Shanghai, China) and cultured according to the protocol. Moreover, the HCT116, DLD1, A549, HeLa, and MDA-MB-231 cells were cultured in DMEM, and H1299 cells were cultured in the RPMI 1640 medium with 10% fetal bovine serum, as per the ATCC guidelines.

### siRNA Knockdown

Non-specific control siRNA and siRNAs for HMOX1, SVCT1, SVCT2, and FBXW7 were purchased from GenePharma (Shanghai, China). The cells were transfected with siRNA oligonucleotides using Lipofectamine 2000 (Invitrogen, CA, USA). The siRNA transfection of cells was performed according to the manufacturer’s instructions. The following siRNAs were used:

si HMOX1: ACAGTTGCTGTAGGGCTTTAT

si SVCT1-1: CATTGAGTCCATCGGAGATTA

si SVCT1-2: CGTGGTGACATCATGGCTATT

si SVCT2-1: GGAAGAAGGGUGUGGGCAA

si SVCT2-2: GGAAAGAGGAAUCCGGAAA

si FBXW7: ACAGGACAGUGUUUACAAATT

### Quantitative RT-PCR Analysis

The total RNA was isolated from the cells by using TRIzol reagent, as per the manufacturer’s instructions. Moreover, the RNA samples were treated with RNase-free DNase and subjected to reverse transcription with PrimerScript RT Reagent Kit. The NanoDrop 2000C Spectrophotometer (Thermo Fisher Scientific, USA) was used to detect the RNA purity and concentration of each sample, while a qRT-PCR analysis was performed in technical triplicate using the TB Green qRT-PCR kit with a Roche LightCycler 96 qRT-PCR system (Roche, Germany). All the data were generated using cDNA from triplicate wells for each condition. Furthermore, the comparative CT method was used to calculate the relative quantity of the target gene messenger RNA (mRNA), and GAPDH gene was used as the internal control. The following procedure was followed for the qRT-PCR experiments: 30 s at 95°C, followed by 40 cycles of 5 s at 95°C and 30 s at 60°C. PCR analysis was performed with human primer sets (forward and reverse, respectively) for Rictor: F’- CGAGTACGAGGGCGGAAT, R’- ATCTGGCCACATTTTGGAGA; HMOX1: F’-TGCTCAACATCCAGCTCTTTGA, R’- AACTGTCGCCACCAGAAAGC; GAPDH: F’- CAACGAATTTGGCTACAGCA, R’- AGGGGTCTACATGGCAACTG.

### Cell apoptosis and cell size assay

The cells were treated with VC for 2 hours and then stained with YF488-Annexin V/PI apoptosis Detection Kit, as per the manufacturer’s protocol. Then, the apoptotic cells were measured by flow cytometry (BD FACSAria III, USA). For the cell size cells assay, the cells were seeded in 6-well plates until reaching 30% confluence, before being cultured under the indicated conditions for 48 hours. Afterward, the cells were harvested and subjected to FACS (BD FA CSAria III, USA) analysis in order to determine cell size. The X-axis indicates the relative cell size.

### Detection of ROS

H1299 cells were seeded in 6-well plates or 24-well plates and were treated with VC for 2 hours. Then, the cells were loaded with the ROS indicator DCF-DA at 37°C for 30 minutes. After wash, the cells in 6-well plates fluorescence intensity were detected by flow cytometry (BD FACSAria III, USA), while the cells in 24-well plates fluorescence intensity were detected by Hybrid Multi-Mode Reader (Bio-Tek, Synergy H1, USA) or fluorescent microscopy (OLYMPUS CKX53, Japan). The DCF-DA fluorescence was determined at an excitation of 485 nm and emission of 538 nm. For the rescue experiments, 2 mM NAC was added in addition to VC.

### Cell viability assays

Cells were seeded in 96-well plates at a concentration of 5 × 10^3^ cells per well with or without VC treatment at the indicated concentrations for the indicated time. Moreover, the cell viability was measured using CCK8, as per the manufacturer’s instructions. Briefly, 10 μL reagent was added to each well and incubated at 37°C for 2–3 hours. Then, the plates were scanned with a plate reader at 450 nm (TECAN, Spark, Switzerland). For rescue experiments, NAC was added in addition to VC.

### Autophagy analysis

Autophagy analysis was performed as previously described [59]. Briefly, the cells were transfected with GFP-LC3 plasmid, and the cells expressing GFP-LC3 were treated with VC for 6 hours. Then, the cells were fixed in 4% paraformaldehyde for 15 minutes. Afterward, the fixed cells were permeabilized using 0.5% Triton X-100 in PBS for 15 minutes and blocked with 1% BSA in PBS for 1 hour at room temperature. Additionally, the nuclei were stained with DAPI. Later, the fluorescence images were captured using laser scanning confocal microscopy (Leica, Germany) in order to visualize the GFP-LC3-containing puncta.

### Colony formation assay

Cells (1000–2000/well) were seeded in 6-well plates and treated with different doses of VC for 2 days, followed by culturing in RPMI-1640 or DMEM medium with 10% FBS for 7 days. Then, the colonies were fixed with 4% paraformaldehyde, washed with PBS, and stained with crystal violet, and each assay was performed in triplicate. In some experiments, NAC was added to the medium in order to eliminate cellular ROS.

### Ubiquitination assay

*In vivo* ubiquitination assays were performed as previously described [60]. Briefly, the transfected cells were treated with 10 μM MG132 for 6 hours before harvesting. Then, the cells were lysed in 60 μL of modified radioimmune precipitation assay buffer (1 × PBS, 1% Nonidet P-40, 0.5% sodium deoxycholate, 1% SDS, 10 mM N-ethylmaleimide, 1 mM phenylmethylsulfonyl fluoride, 1 mM NaV_3_O_4_, and 1 mM NaF). Afterward, thecCell lysates were boiled for 10 minutes, diluted in 10 volumes of lysis buffer without SDS, and subjected to immunoprecipitation using 4 μg of anti-Rictor antibody. Later, the ubiquitination of Rictor was detected by immunoblotting.

### Cycloheximide (CHX) Chase Assay

In order to assess the effect of VC on Rictor protein stability, the cells were incubated with 20 μg/mL CHX for 6 hours to inhibit *de novo* protein synthesis, before being treated with VC. At the indicated time points, the cell lysates were harvested and subjected to western blot analysis by using the indicated antibodies.

### SUnSET Measurements

In order to assess the effect of VC on protein synthesis, the cells were incubated with 1 μM puromycin with VC with or without added NAC for 6 hours. At the indicated time points, CHX was added and subjected to western blot analysis by using the indicated antibodies.

### RNA-seq and DEG Identification

The RNA samples were collected from the VC (n = 3) and control (n = 3) groups, respectively. The cDNA libraries were sequenced on the Illumina sequencing platform by Genedenovo Biotechnology Co., Ltd (Guangzhou, China). The data obtained by sequencing were called raw reads or raw data. Then, quality reads (QC) were performed on the raw reads in order to determine whether the sequencing data were suitable for subsequent analysis. After the quality control was passed, the clean reads, after filtering, were compared to the reference genome by using hierarchical indexing for spliced alignment of transcripts (HISAT). As regards DEGs, we utilized the limma package with an adjustment. Additionally, the DEGs were defined as those genes with a |log2 fold change| > 1 and adjusted *p*-value < 0.05 as the cut-off criteria. Besides, the adjusted *p*-value using the Benjamini and the Environ Sci Pollut Res Hochberg false discovery rate (FDR) method was applied to correct for the occurrence of false positive results.

### Immunoblotting

The cells were harvested and suspended in the radio immune precipitation assay (RIPA) buffer on ice for 30 minutes. Moreover, the proteins were separated by sodium dodecyl sulphate-polyacrylamide gel electrophoresis (SDS-PAGE) and transferred onto the nitrocellulose (NC) membrane (0.45 μm, GE). Then, the membranes were probed with the primary and secondary antibodies. The primary antibody used were Actin (1:3000; 20536-1-AP; Proteintech), LC3II (1:1000; 18725-1-AP; Proteintech), p62 (1:1000; 18420-1-AP; Proteintech), HMOX1 (1:1000; 10701-1-AP; Proteintech), FBXW7α (1:1000; 28424-1-AP; Proteintech), pT389-S6K (1:1000; 9234S/L; Cell Signaling Technology), p-S6 (1:1000; 4858S; Cell Signaling Technology), S6K (1:1000; 9202S; Cell Signaling Technology), S6 (1:1000; 2217S; Cell Signaling Technology), Rictor (1:1000; 9476; Cell Signaling Technology), pS473-AKT (1:1000; 9271; Cell Signaling Technology), AKT (1:1000; 9272; Cell Signaling Technology), p4EBP1 (1:1000; 9451S; Cell Signaling Technology), anti-Puromycin (1:1000, EQ0001, Kerafast), SVCT1 (1:1000; NBP2-13318; Novus), SVCT2 (1:1000; NBP2-13319; Novus), AMPK (1:1000; 2532S; Cell Signaling Technology), p-AMPK (1:1000; 2535S; Cell Signaling Technology), p38 (1,1000; 14064-1-AP; Proteintech), p-p38 (1,1000; 9211S; Cell Signaling Technology), and GAPDH (1,2000; db106; Hangzhou Bio Technolog), while the secondary antibodies were obtained from Sigma. After being incubated with primary antibody overnight, the membranes were incubated with a secondary antibody conjugated with horseradish peroxidase (HRP) at room temperature for 1 hour. Finally, the proteins were detected by using the imaging system (Bio-Rad, Hercules, CA, USA). The ImageJ software was used to quantify the protein abundance.

### Xenografts

In order to test the effect of VC, approximately 5 × 10^6^ cells in 100 μL were injected into the flank of 6-to-8-week-old female athymic nude mice. After 14 days, the mice with tumors of 40–60 mm^3^ were randomly divided into two groups. The tumors were obtained on the indicated day after injection. The tumor sizes were measured with electronic calipers, and the volumes were calculated using the formula in an unblinded manner: (L x W2) x 0.5, where L denotes length and W denotes width.

### Measurement of VC Content

H1299 cells were plated in a 6-well plate and reached 70–80% confluence before VC treatment. Afterward, the cells were washed twice with ice-cold PBS and then extracted with ice-cold 70% (v/v) methanol, 30% solution A (10 mM-tetrabutylammonium hydrogen sulphate, 10 mM-KH_2_PO_4_, 0.5%-methanol; pH 6.0) containing 1 mM-EDTA for 10 minutes at 0°C. Later, the samples were centrifuged at 10000 g for 20 minutes at 4°C, and the supernatant was collected and placed at -80°C for subsequent analysis. Finally, the concentration of VC was measured by high-performance liquid chromatographic with the UV detection wavelength set at 265 nm, in accordance with the work of Catia et al [61].

### Statistical analysis

The GraphPad Prism 9.0 software was used for data analysis. The data were presented as mean ± SEM. The statistical tests included unpaired one-tailed or two-tailed Student’s t-test and one-way analysis of the variance, and a *p* value of 0.05 was considered statistically significant. In the graphed data, *, **, and *** denote the *p* values of < 0.05, 0.01, and 0.001, respectively, and ns = not significant.

## Supporting information

S1 Fig Pharmacological VC Inhibits mTOR Activity. (A, B) HeLa and MDA-MB-231 cells were treated with the indicated concentrations of VC for 2 h, the indicated proteins were detected by WB (A), and quantified by ImageJ, n = 3 (B). (C, D) H1299 cells treated with the indicated concentrations (C) or time (D) of VC, the intracellular VC content by high-performance liquid chromatography, n = 3. (E) HCT116 cells was treated with indicated concentrations VC for 48 h, and cell viability was detected by CCK-8, n = 3. (F) HCT116 cells was treated with indicated concentrations of VC, and cell viability was detected by clone formation. (G) HCT116 cells were treated with indicated concentrations of VC for 6 h after which autophagy was analyzed by examining p62 levels. (H, I) HCT116 cells was treated with Bafilomycin A1 and 1.0 mM VC for 6 h, the cell autophagy was analyzed by examining the GFP-LC3 puncta (H). The quantified data of GFP-LC3 puncta were present (I). (J-M) H1299 cells (J, K) and HCT116 cells (L, M) was treated with 1.0 mM VC for 2 h, and cell apoptosis was detected by FACS. Data were analyzed by one-way ANOVA (B-E) or t-test (I, K, M), *p* value was considered statistically significant, ** denote *p* values of < 0.01, *** denote *p* values of < 0.001.
(TIF)

S2 Fig Pharmacological VC Affects Cell viability through Inhibiting mTOR Activation. (A) H1299 cells were treated with 1.0 mM VC and Torin1 for 48 h, after which cell viability was assessed by CCK-8, n = 3. (B) H1299 cells were treated with 1.0 mM VC and MHY1485 for 48 h, after which cell viability was assessed by CCK-8 assay, n = 3. (C) H1299 cells were injected into nude mice subcutaneous and the mice weight was measured. Data were analyzed by two-way ANOVA, *p* value was considered statistically significant, * denote *p* values of < 0.05, *** denote *p* values of < 0.001, ns denote not significant.
(TIF)

S3 Fig SVCT2 Mediates the Uptake of VC to Inhibit the mTOR Pathway. (A) H1299 cells were stimulated with insulin for the indicated time after pretreatment with 0.5 mM VC for 2 h, the indicated proteins were detected by WB. (B) H1299 cells were stimulated with amino acids (AA) for the indicated time after pretreatment with 0.5 mM VC for 2 h, the indicated proteins were detected by WB. (C) Knockdown the p38 H1299 cell was treated with the indicated concentrations of VC for 2 h, the indicated proteins were detected by WB. (D) H1299 cell was stimulated with insulin for 15 min after pretreatment of with VC and/or the p38 MAPK inhibitor SB203580, the indicated proteins were detected by WB. (E) Knockdown the AMPK H1299 cell was treated with the indicated concentrations of VC for 2 h, the indicated proteins were detected by WB. (F) H1299 cell was stimulated with insulin for 15 min after pretreatment of with VC and/or the AMPK inhibitor Compound C for 2 h, the indicated proteins were detected by WB. (G) H1299 cell was treated with the indicated concentrations of VC and/or the GLUT1 inhibitor STF-31 for 2 h, the indicated proteins were detected by WB. (H) H1299 cell was stimulated with insulin for 15 min after pretreatment of with VC and/or the GLUT1 inhibitor STF-31 for 2 h, the indicated proteins were detected by WB. (I) Knockdown the SVCT1 in H1299 cells, and cells were treated with indicated concentrations VC for 2 h, and the indicated proteins were detected by WB. (J) SVCT2-depleted H1299 cells were stimulated with insulin for 30 min after pretreatment with 0.5 mM and 1.0 mM VC for 2 h, the indicated proteins were detected by WB.
(TIF)

S4 Fig Pharmacological VC to Inhibit the mTOR Pathway in a ROS-dependent Manner. (A, B) H1299 cells was treated with VC alone or in combination with 2.0 mM NAC, and the ROS of cells was detected by microplate-reader (A), the quantified data of Fluorescence intensity were present, n = 3 (B). (C) HCT116 cells were treated with the indicated concentrations of VC alone or in combination with 2.0 mM NAC for 2 h, and the indicated proteins were detected by WB. (D) HCT116 cells were treated with 1.0 mM VC alone or in combination with 2.0 mM NAC, and then cell viability was assessed by CCK-8, n = 3. Data were analyzed one-way ANOVA (B, D), *p* value was considered statistically significant, ** denote *p* values of < 0.01, *** denote *p* values of < 0.001.
(TIF)

S5 Fig Pharmacological VC Inhibits the mTORC1 Pathway by Promoting the Degradation of Rictor. (A) The expression of Rictor gene was examined by qRT-PCR, n = 3. (B, C) H1299 cells were treated with VC or cycloheximide (CHX) for the indicated time, and then WB was used to evaluate the levels of the indicated proteins (B). Quantitative data for Rictor protein level was presented, n = 3 (C). (D, E) H1299 cells were treated with the indicated concentrations of VC or MG132 for 6 h, after which WB was used to evaluate the levels of the indicated proteins (D). Quantitative data for Rictor protein level was presented, n = 3 (E). (F, G) H1299 cells were treated with the indicated concentrations of VC alone or in combination with LiCl for 6 h, and then the level of Rictor was evaluated by WB (F). Quantitative data of Rictor/Actin was presented, n = 3 (G). (H, I) H1299 cells were treated with the indicated concentrations of VC alone or in combination with LiCl for 2 h, and the indicated proteins were detected by WB (H). Quantitative data was presented, n = 3 (I). (J) H1299 cells were treated with 0.5 mM VC alone or in combination with LiCl for 48 h, and then cell viability was assessed by CCK-8, n = 3. Data were analyzed by two-way ANOVA (C, E, G, I, J) or t-test (A), *p* value was considered statistically significant, * denote *p* values of < 0.05, ** denote *p* values of < 0.01, *** denote *p* values of < 0.001, ns denote not significant.
(TIF)

S6 Fig Pharmacological VC inhibits the mTORC1 pathway by inducing HMOX1 expression. (A) The expression of HMOX1 was detected by qPCR, n = 3. (B, C) H1299 cells were treated with the indicated concentrations of VC alone or in combination with ZnPPIX, and the indicated proteins were detected by WB (B). Quantitative data was presented, n = 3 (C). (D) The viability of ZnPPIX-treated H1299 cells was assessed by CCK-8 assay, n = 3. (E, F) The levels of autophagy in ZnPPIX treated H1299 cells were analyzed by examining GFP-LC3 puncta (E). Quantitative data for the GFP-LC3 puncta are present (F). Data were analyzed by two-way ANOVA (C, D) or t-test (A, F)), *p* value was considered statistically significant, ** denote *p* values of < 0.01, *** denote *p* values of < 0.001, ns denote not significant.
(TIF)
