# Supplementary figures and images for: Pharmacological vitamin C inhibits mTOR signaling and tumor growth by degrading Rictor and inducing HMOX1 expression
Source: PLoS Genet. 2023 Feb 14;19(2):e1010629. doi: 10.1371/journal.pgen.1010629 (PMC9928125; doi:10.1371/journal.pgen.1010629)

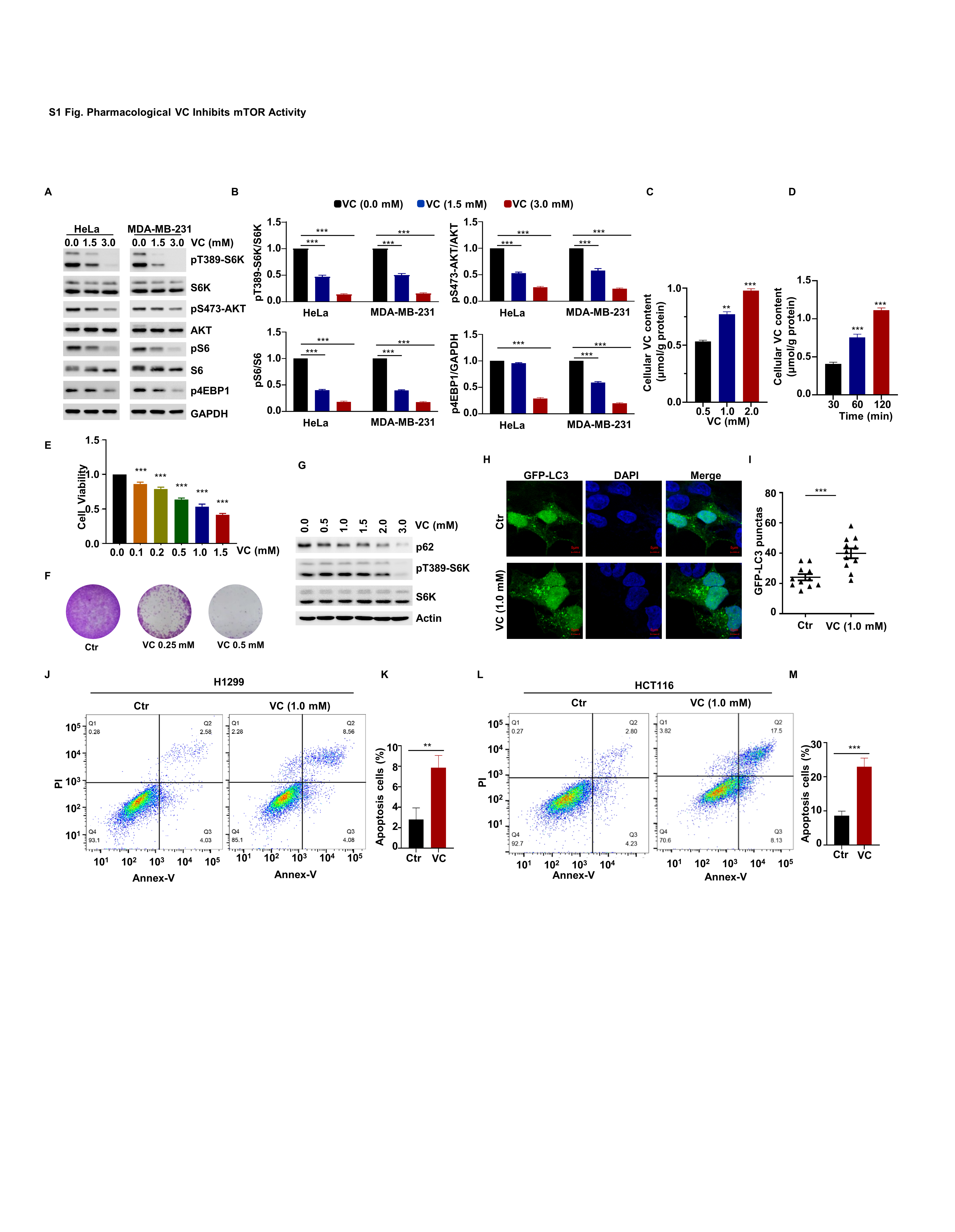

Supplement: S1 Fig — (A, B) HeLa and MDA-MB-231 cells were treated with the indicated concentrations of VC for 2 h, the indicated proteins were detected by WB (A), and quantified by ImageJ, n = 3 (B). (C, D) H1299 cells treated with the indicated concentrations (C) or time (D) of VC, the intracellular VC content by high-performance liquid chromatography, n = 3. (E) HCT116 cells was treated with indicated concentrations VC for 48 h, and cell viability was detected by CCK-8, n = 3. (F) HCT116 cells was treated with indicated concentrations of VC, and cell viability was detected by clone formation. (G) HCT116 cells were treated with indicated concentrations of VC for 6 h after which autophagy was analyzed by examining p62 levels. (H, I) HCT116 cells was treated with Bafilomycin A1 and 1.0 mM VC for 6 h, the cell autophagy was analyzed by examining the GFP-LC3 puncta (H). The quantified data of GFP-LC3 puncta were present (I). (J-M) H1299 cells (J, K) and HCT116 cells (L, M) was treated with 1.0 mM VC for 2 h, and cell apoptosis was detected by FACS. Data were analyzed by one-way ANOVA (B-E) or t-test (I, K, M), p value was considered statistically significant, ** denote p values of < 0.01, *** denote p values of < 0.001. (TIF) [file pgen.1010629.s001.TIF]

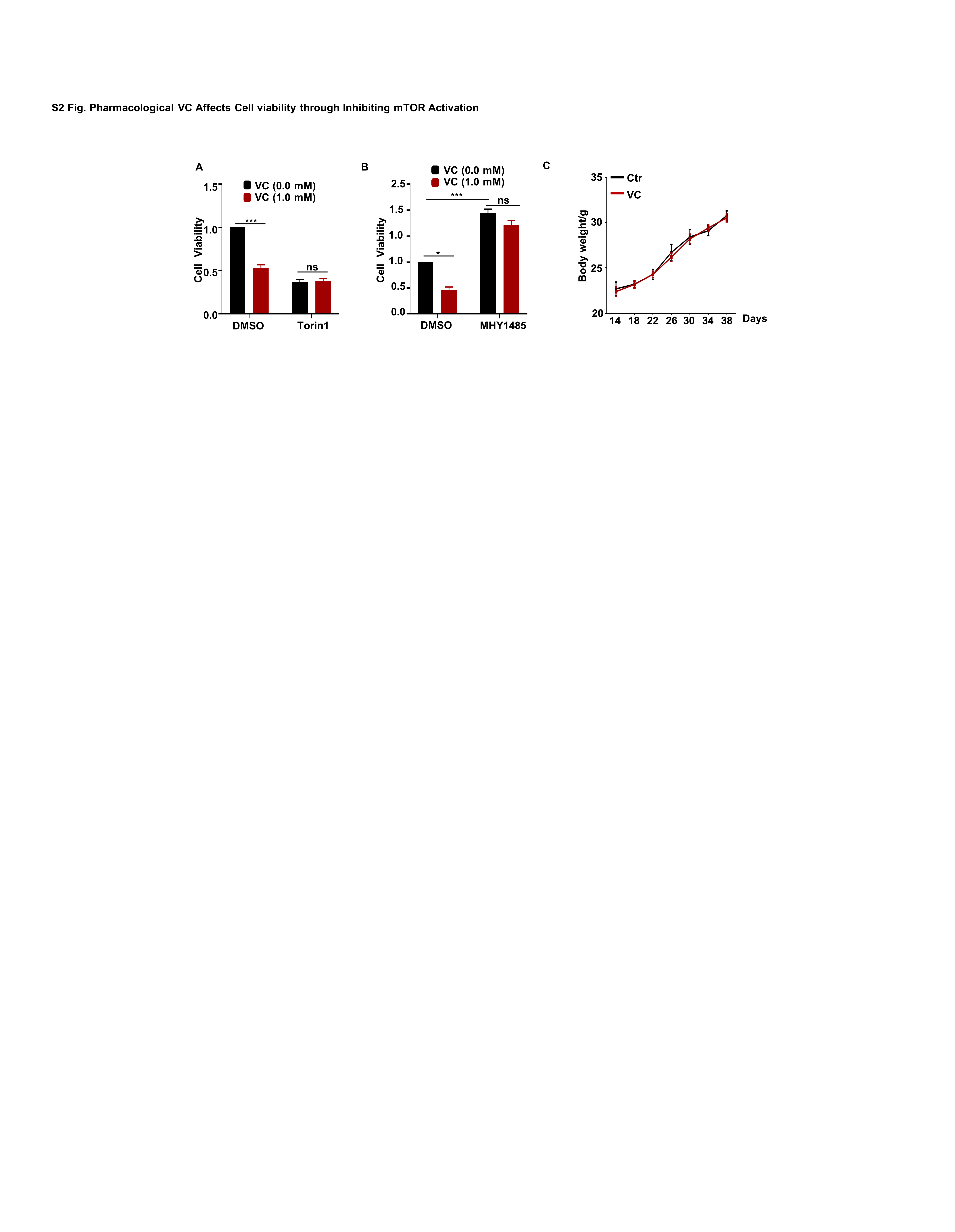

Supplement: S2 Fig — (A) H1299 cells were treated with 1.0 mM VC and Torin1 for 48 h, after which cell viability was assessed by CCK-8, n = 3. (B) H1299 cells were treated with 1.0 mM VC and MHY1485 for 48 h, after which cell viability was assessed by CCK-8 assay, n = 3. (C) H1299 cells were injected into nude mice subcutaneous and the mice weight was measured. Data were analyzed by two-way ANOVA, p value was considered statistically significant, * denote p values of < 0.05, *** denote p values of < 0.001, ns denote not significant. (TIF) [file pgen.1010629.s002.TIF]

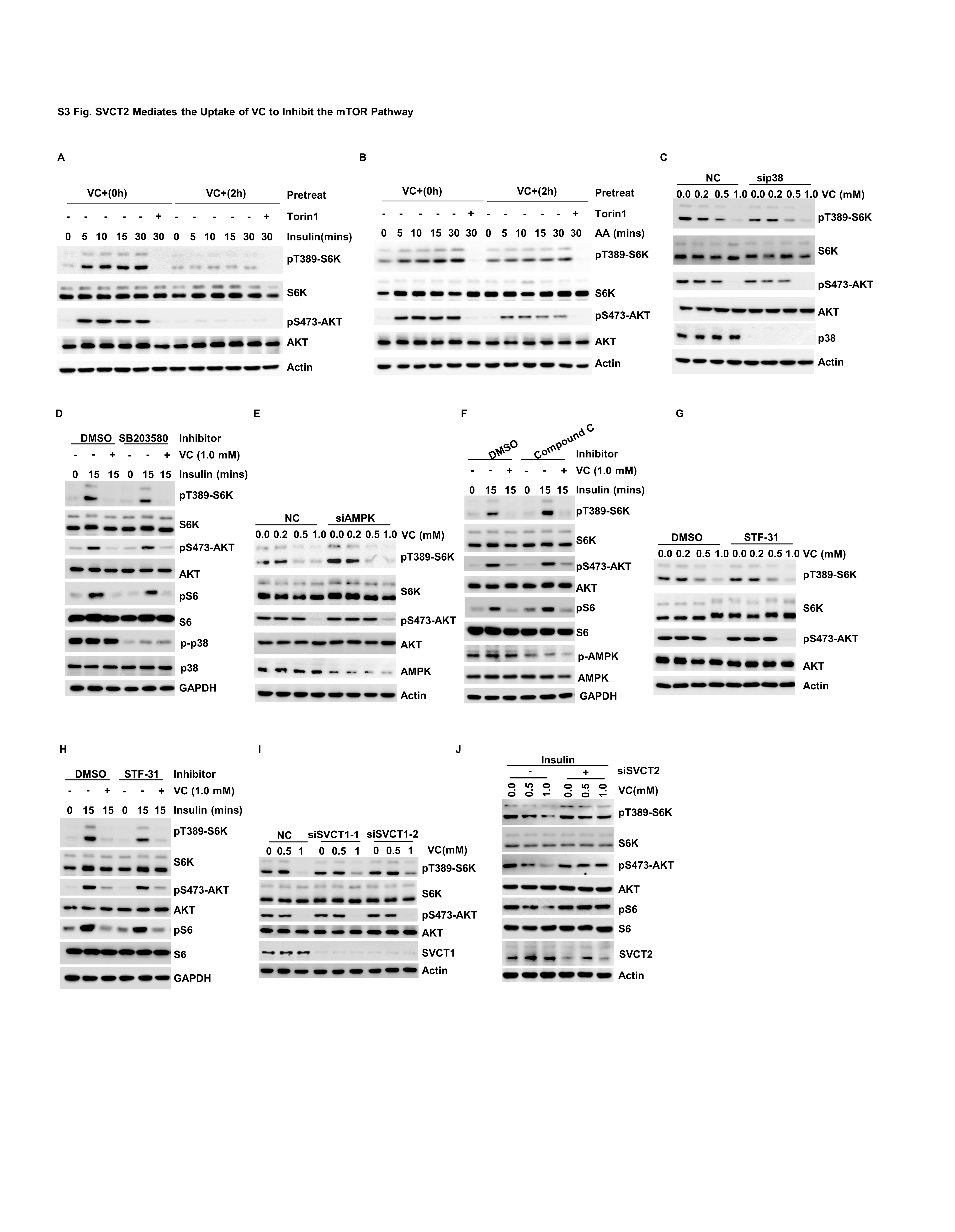

Supplement: S3 Fig — (A) H1299 cells were stimulated with insulin for the indicated time after pretreatment with 0.5 mM VC for 2 h, the indicated proteins were detected by WB. (B) H1299 cells were stimulated with amino acids (AA) for the indicated time after pretreatment with 0.5 mM VC for 2 h, the indicated proteins were detected by WB. (C) Knockdown the p38 H1299 cell was treated with the indicated concentrations of VC for 2 h, the indicated proteins were detected by WB. (D) H1299 cell was stimulated with insulin for 15 min after pretreatment of with VC and/or the p38 MAPK inhibitor SB203580, the indicated proteins were detected by WB. (E) Knockdown the AMPK H1299 cell was treated with the indicated concentrations of VC for 2 h, the indicated proteins were detected by WB. (F) H1299 cell was stimulated with insulin for 15 min after pretreatment of with VC and/or the AMPK inhibitor Compound C for 2 h, the indicated proteins were detected by WB. (G) H1299 cell was treated with the indicated concentrations of VC and/or the GLUT1 inhibitor STF-31 for 2 h, the indicated proteins were detected by WB. (H) H1299 cell was stimulated with insulin for 15 min after pretreatment of with VC and/or the GLUT1 inhibitor STF-31 for 2 h, the indicated proteins were detected by WB. (I) Knockdown the SVCT1 in H1299 cells, and cells were treated with indicated concentrations VC for 2 h, and the indicated proteins were detected by WB. (J) SVCT2-depleted H1299 cells were stimulated with insulin for 30 min after pretreatment with 0.5 mM and 1.0 mM VC for 2 h, the indicated proteins were detected by WB. (TIF) [file pgen.1010629.s003.TIF]

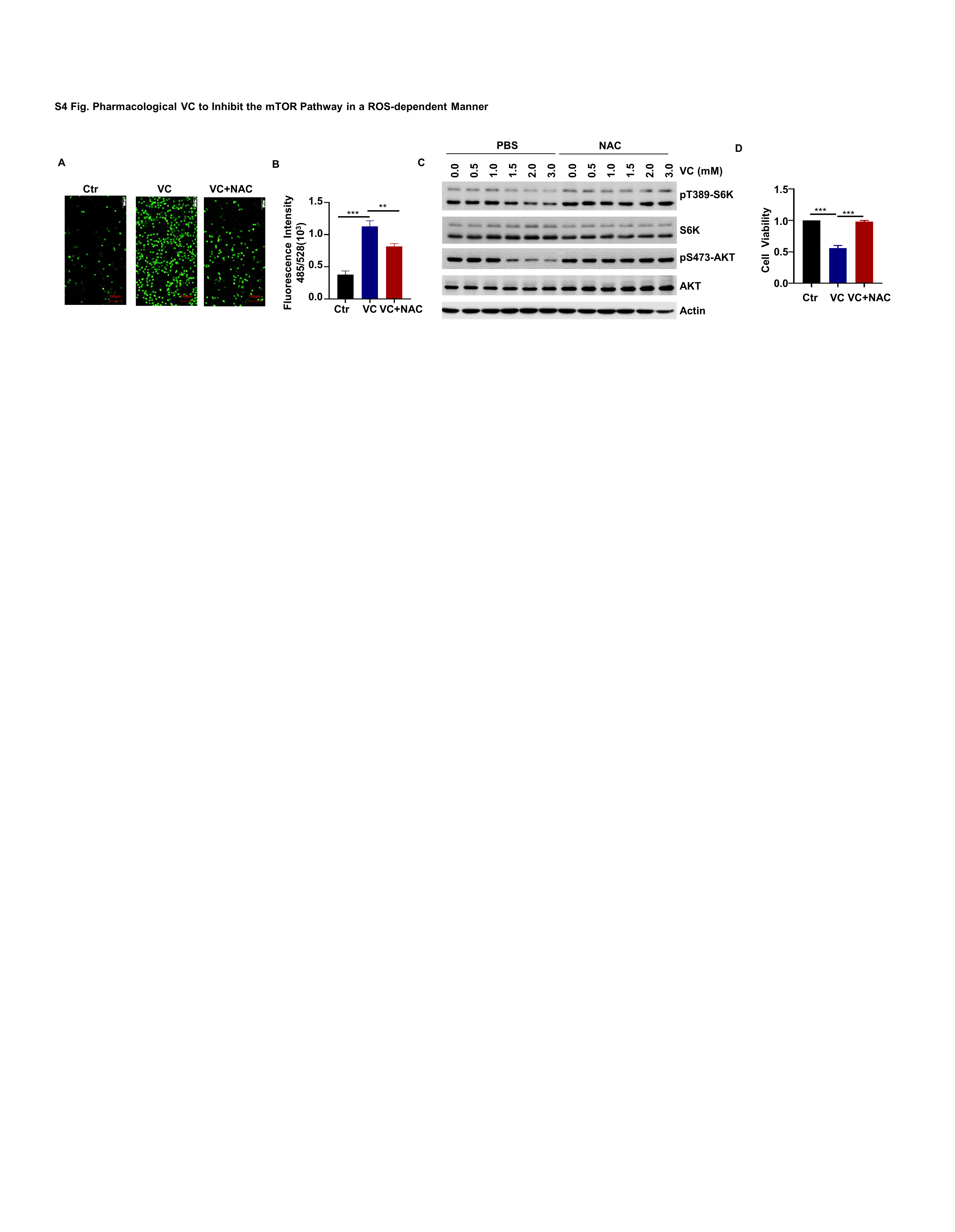

Supplement: S4 Fig — (A, B) H1299 cells was treated with VC alone or in combination with 2.0 mM NAC, and the ROS of cells was detected by microplate-reader (A), the quantified data of Fluorescence intensity were present, n = 3 (B). (C) HCT116 cells were treated with the indicated concentrations of VC alone or in combination with 2.0 mM NAC for 2 h, and the indicated proteins were detected by WB. (D) HCT116 cells were treated with 1.0 mM VC alone or in combination with 2.0 mM NAC, and then cell viability was assessed by CCK-8, n = 3. Data were analyzed one-way ANOVA (B, D), p value was considered statistically significant, ** denote p values of < 0.01, *** denote p values of < 0.001. (TIF) [file pgen.1010629.s004.TIF]

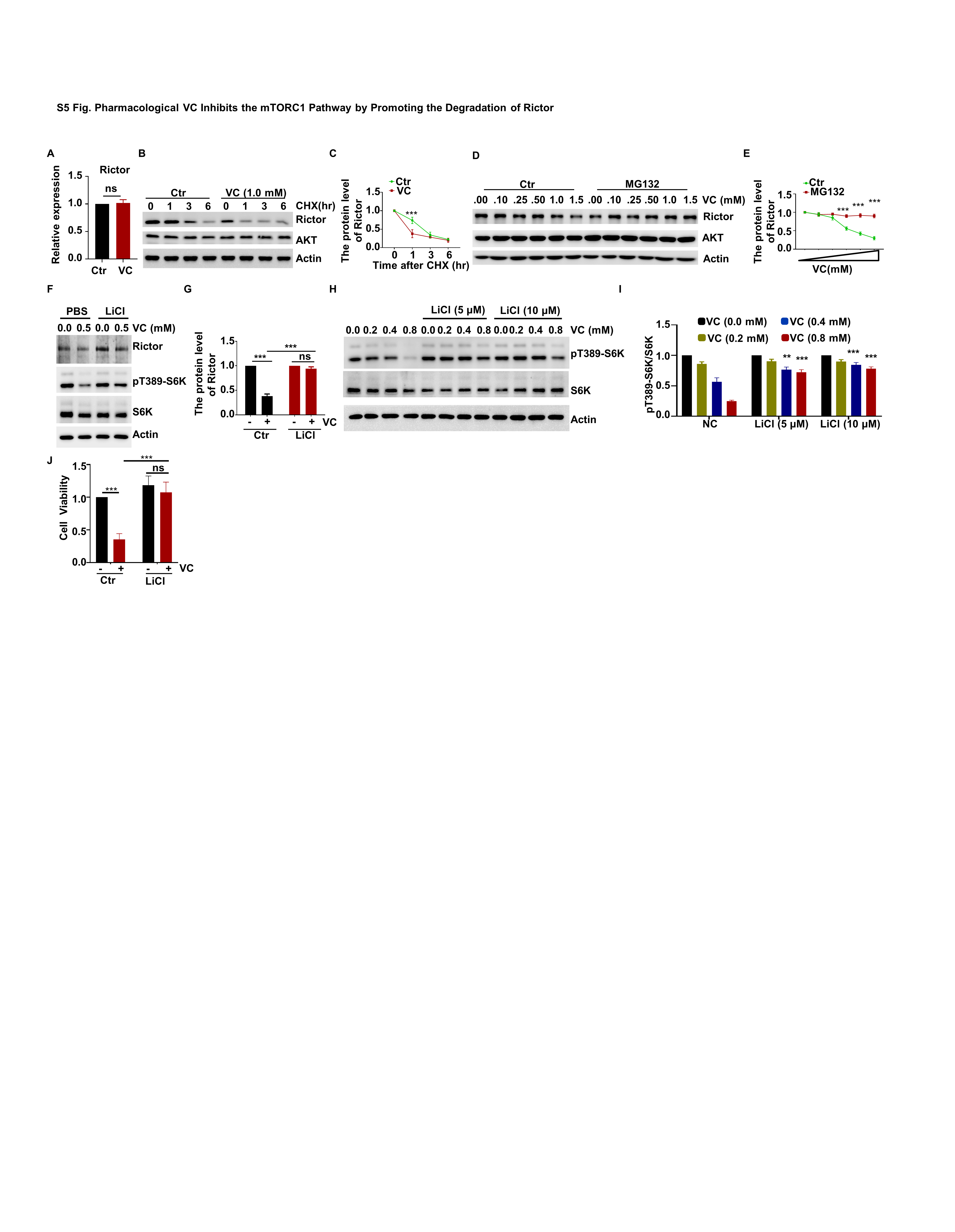

Supplement: S5 Fig — (A) The expression of Rictor gene was examined by qRT-PCR, n = 3. (B, C) H1299 cells were treated with VC or cycloheximide (CHX) for the indicated time, and then WB was used to evaluate the levels of the indicated proteins (B). Quantitative data for Rictor protein level was presented, n = 3 (C). (D, E) H1299 cells were treated with the indicated concentrations of VC or MG132 for 6 h, after which WB was used to evaluate the levels of the indicated proteins (D). Quantitative data for Rictor protein level was presented, n = 3 (E). (F, G) H1299 cells were treated with the indicated concentrations of VC alone or in combination with LiCl for 6 h, and then the level of Rictor was evaluated by WB (F). Quantitative data of Rictor/Actin was presented, n = 3 (G). (H, I) H1299 cells were treated with the indicated concentrations of VC alone or in combination with LiCl for 2 h, and the indicated proteins were detected by WB (H). Quantitative data was presented, n = 3 (I). (J) H1299 cells were treated with 0.5 mM VC alone or in combination with LiCl for 48 h, and then cell viability was assessed by CCK-8, n = 3. Data were analyzed by two-way ANOVA (C, E, G, I, J) or t-test (A), p value was considered statistically significant, * denote p values of < 0.05, ** denote p values of < 0.01, *** denote p values of < 0.001, ns denote not significant. (TIF) [file pgen.1010629.s005.TIF]

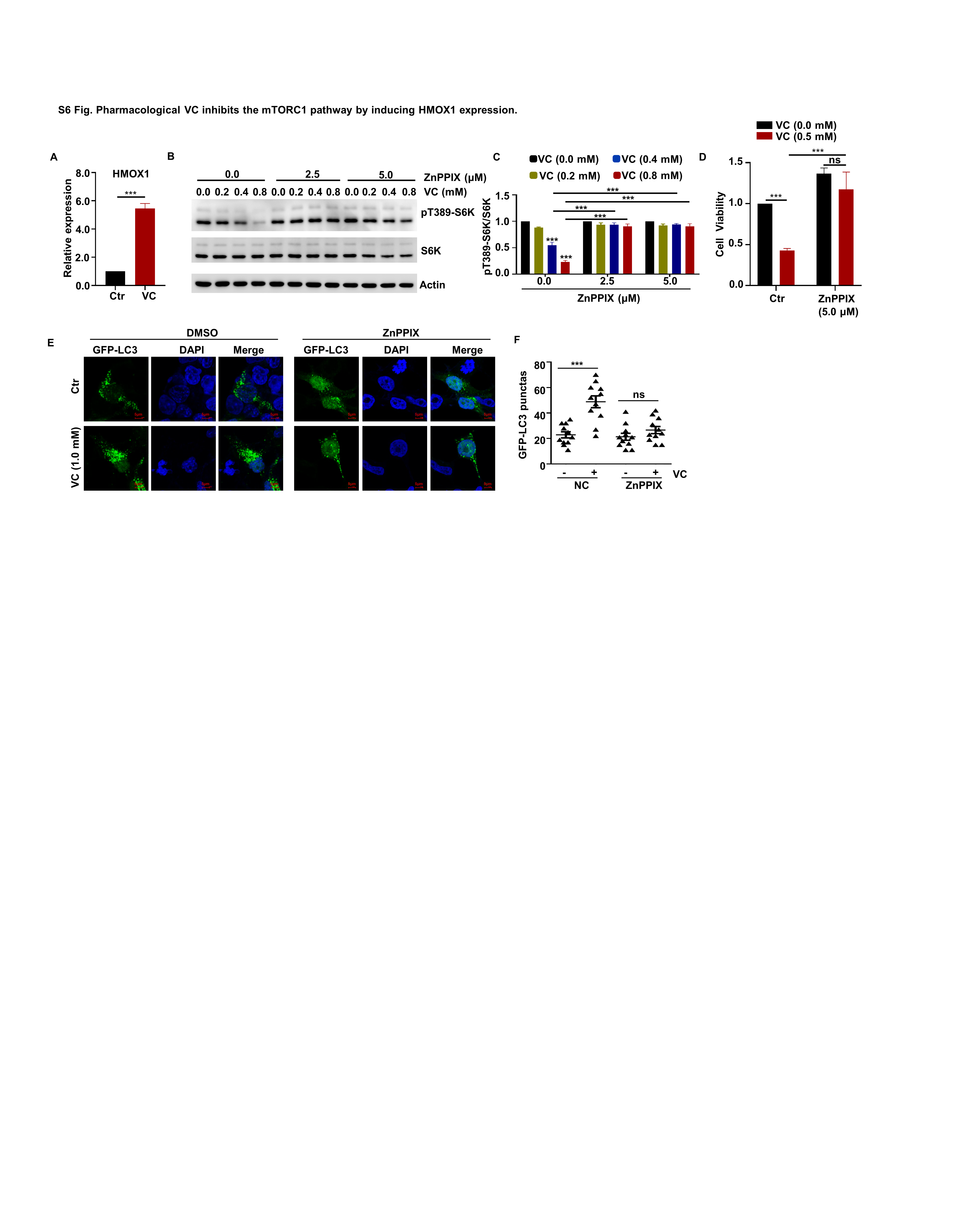

Supplement: S6 Fig — (A) The expression of HMOX1 was detected by qPCR, n = 3. (B, C) H1299 cells were treated with the indicated concentrations of VC alone or in combination with ZnPPIX, and the indicated proteins were detected by WB (B). Quantitative data was presented, n = 3 (C). (D) The viability of ZnPPIX-treated H1299 cells was assessed by CCK-8 assay, n = 3. (E, F) The levels of autophagy in ZnPPIX treated H1299 cells were analyzed by examining GFP-LC3 puncta (E). Quantitative data for the GFP-LC3 puncta are present (F). Data were analyzed by two-way ANOVA (C, D) or t-test (A, F)), p value was considered statistically significant, ** denote p values of < 0.01, *** denote p values of < 0.001, ns denote not significant. (TIF) [file pgen.1010629.s006.TIF]
